# Using a virtual reality role-playing game to enhance interaction dynamics and improve social attitudes toward transgender people

**DOI:** 10.1038/s41598-026-46402-3

**Published:** 2026-04-10

**Authors:** Cassandra L. Crone, Rachel W. Kallen, Michael J. Richardson

**Affiliations:** School of Psychological Sciences, Faculty of Medicine, Health and Human Sciences, Macquarie University, Sydney, NSW Australia

**Keywords:** Health care, Psychology, Psychology

## Abstract

While virtual reality (VR) interventions have demonstrated efficacy in reducing intergroup prejudice, they have yet to be applied to transprejudice. Accordingly, we developed a VR role-playing game (RPG) that integrates perspective-taking and intergroup-contact for transgender identities. We evaluated the RPG’s impact on cisgender individuals’ movement dynamics and social attitudes toward transgender people using a mixed experimental design, in which 162 participants embodied a self-customised transgender avatar and interacted with a cisgender or transgender virtual colleague. Empathy increased throughout the interaction, consistent with mediating the relationship between virtual embodiment and post-session gender essentialism, but only those partnered with a transgender colleague reported increased within-session interpersonal affiliation. Aligning with shifts in empathy, participants’ postural dynamics became less constrained and more complex over time, although women adapted more readily to the RPG than men. Taken together, our findings highlight the potential value of simulated interactions that promote rapport-building between cisgender and transgender individuals.

**Supplementary Information:**

The online version contains supplementary material available at 10.1038/s41598-026-46402-3.

## Introduction

Against the backdrop of an evolving socio-political landscape, rising backlash to gender equality, and narratives that debate the legitimacy of transgender and gender diverse (TGD) identities, there is an increasingly urgent need to challenge the pervasiveness of gender essentialism (i.e., beliefs that gender is binary, discrete, immutable, and biologically based)^1^. Such beliefs contribute to gender inequities in education, healthcare, and the workplace^2–4^ for both TGD people and cisgender women alike. Moreover, those who endorse gender essentialist beliefs are also more likely to favour cisgender individuals, enact delegitimising microaggressions, engage in gender identity denial, and stereotype TGD people based on their assigned sex at birth^5^. To reduce transprejudice within everyday interactions, there is a critical demand for interventions that go beyond traditional approaches.

Recent research has found that virtual reality (VR) interventions—namely, VR perspective taking (VRPT) and VR-mediated contact—can effectively reduce intergroup prejudices^6^. The former, VRPT, adopts an individual strategy, whereby participants virtually embody an avatar representing a marginalised outgroup identity to simulate experiencing the world from another’s perspective^7^. The latter, VR-mediated contact, adopts an intergroup strategy, whereby participants synchronously interact with an outgroup member to simulate positive, direct intergroup contact^8^. Thus far, no research has applied either approach to TGD identities. In the current study, we investigate the impact of transgender VRPT and VR-mediated cisgender-transgender contact on gender essentialism, intergroup empathy, and interpersonal affiliation.

Despite well-documented evidence of anti-transgender stigma in society, there is limited research on cisgender-transgender contact, overall. Much of the current body of knowledge relies on cross-sectional, survey-based, and correlational evidence, suggesting that self-reported contact with TGD people is associated with reduced levels of transphobia and transprejudice^9^. Some experimental paradigms have echoed these findings using imagined contact^10^, parasocial, vicarious contact^11^, and direct contact via teaching interventions^12^, finding that some contact with transgender outgroup targets is better than no contact at all for cisgender individuals. Although interventions based on direct contact experiences are expected to exhibit the strongest effects^13^, in-person encounters between cisgender and transgender people are limited and face several barriers (e.g., sparse minority population, intergroup anxiety and avoidance, potential for harm)^14^. Thus, we highlight a need to embrace more immersive simulations of contact when developing interventions aimed at reducing transprejudice among cisgender individuals.

Only one study, to date, has employed an immersive, real-time simulation of contact, finding that online, text-based interaction with a transgender woman reduced transgender stigma, but only for cisgender men^15^. Text-based contact, however, lacks the visual and embodied cues that are present during real-world interpersonal interactions. Recent research has demonstrated that fully immersive interactions undertaken in VR result in greater empathy, prosocial behaviour, and prejudice reduction toward marginalised outgroup targets, in comparison to such simulations undertaken online^16,17^. Notably, this relies on embodied perspective taking, whereby participants experience a sense of ownership and agency over a virtual avatar representing a marginalised outgroup identity (i.e., VRPT)^18,19^. Mere exposure to such embodiment, however, is insufficient to induce attitudinal or behavioural changes^20,21^. Rather, successful VRPT requires that the content of the interaction be ecologically valid, more closely approximating real-world contexts in which intergroup prejudices arise^22^.

Taken together, VR-mediated contact and VRPT may serve to improve cisgender-transgender relations by enhancing the affective (e.g., empathy) and cognitive (e.g., knowledge acquisition, familiarity) mechanisms associated with prejudice reduction^23^. Empathy, in particular, is thought to underlie the relationship between virtual immersion and positive change^24^. Thus, we designed a VR role-playing game (RPG), in which the narrative progressively increased rapport-building between cisgender participants and either a transgender or cisgender interaction partner. Not only does such an approach afford an ecologically valid simulation of positive contact during VRPT, but also it provides a novel method of capturing subtle information that an individual might reveal through non-verbal behaviours, such as postural activity, in real time.

Postural activity, or more specifically *postural sway*, refers to the subtle shifts or fluctuations in movement that persist when standing upright, which are functionally critical to exploring the information in one’s environment^25^. This variability in postural sway systematically fluctuates over time, exhibiting meaningful structure that allows individuals to adapt to varying personal and task-relevant constraints^26,27^. An individual’s postural activity, thus, provides an embodied measure of the codependent, non-verbal, and linguistic processes that characterise direct, face-to-face social interactions^28,29^. Changes in situational constraints, as well as an individual’s intentional state, can significantly influence the structural complexity of postural fluctuations^30,31^. In the current study, we examine the dynamics of postural behaviours exhibited throughout the RPG simulation to explore how VR-mediated contact and VRPT are embodied. Given that such behaviour is not a static representation of mental states, but rather, an emergent property of the complex interactions between brain, body, and environment^32^, this approach can reveal which situational events, in particular, might contribute to adaptation as a result of undertaking the RPG.

We expected that exposure to the RPG simulation would be associated with increased empathy and interpersonal affiliation with transgender outgroup targets, alongside lower endorsement of gender essentialist beliefs. Additionally, we hypothesised that empathy would play a mediating role in the relationships between virtual embodiment and these outcomes. However, no research, to date, has evaluated both VR-mediated contact and VRPT in the context of transprejudice among cisgender people. Using a mixed experimental design, we investigated the impact of our virtual RPG on the self-reported attitudes (i.e., gender essentialism, empathy, affiliation) and non-verbal behaviours (i.e., postural sway) of cisgender individuals, who embodied a self-customised transgender avatar while interacting with either a transgender or cisgender colleague.

## Results

**Fig. 1:**
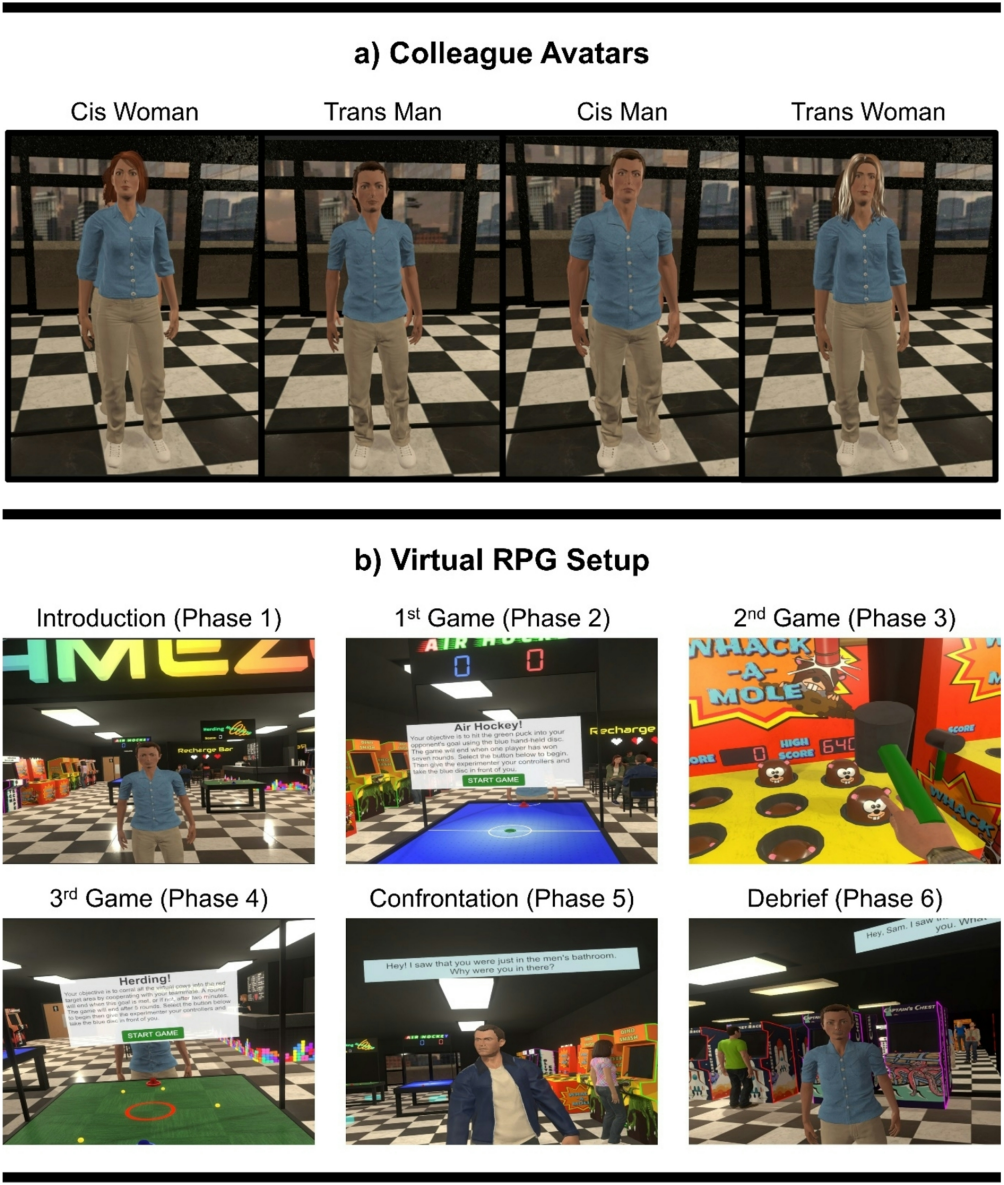
(**a**) Participants were randomly assigned to interact with either a transgender or cisgender virtual avatar, such that women were paired with either a cisgender woman or transgender man (left), whereas men were paired with a cisgender man or transgender woman (right). (**b**) Progression through the RPG narrative occurred across six discrete phases, as displayed here. Postural activity was recorded across each phase, while state empathy was recorded following the first, second, and third games, and following the debrief.

We designed the RPG as an after-work team-building event set in an arcade-style gaming centre to serve as a model of everyday interaction in which transprejudice may emerge. All participants undertook VRPT by embodying a transgender avatar within a structured series of scripted, rapport-building, narrative scenarios, while those who interacted with a transgender colleague additionally experienced VR-mediated contact (see Fig. 1a). Postural activity timeseries were recorded across six discrete phases of the RPG (displayed in Fig. 1b) and assessed within-subjects using detrended fluctuation analysis (DFA). We then used a generalised linear mixed model (GLMM) to assess whether changes in postural activity observed throughout the RPG differed based on the colleague’s gender (i.e., VR-mediated contact) as well as between women and men participants. Next, we examined differences by colleague and participant gender for all self-report measures, as recorded within- (i.e., state empathy, affiliation) and between-subjects (i.e., trait empathy, social desirability, embodiment, gender essentialism). Finally, we used a series of mediation analyses to assess indirect pathways from perceived virtual embodiment to post-session gender essentialism and within-session change in interpersonal affiliation via state empathy toward the virtual colleague. Further details on the research design are outlined in the Methods section.

Figure 1a and b were composed by the authors using screenshots of the virtual environment, also developed by the authors and hosted in Unity (version 2020.3.42f1).

### Postural movement dynamics reveal reduced behavioural constraint over time

We used DFA to quantify the degree of self-similarity within participants’ postural sway timeseries across the six stages of the RPG (see Methods for a detailed description of data processing). DFA determines the average magnitude of residual variance across a range of window sizes within a given timeseries (i.e., segments of 8, 16, 32, 64, 128, 256, … data points) and then plots the average residual variance estimates (i.e., root mean square) as a function of window size in log–log form^32^. The slope of the regression line fitting this plot is denoted as *α* (alpha), which provides an estimate of the fractal, or self-similar structure within participants’ postural sway timeseries. Namely, *α* characterises the stochastic nature of this fractal structure, such that *α* values closer to 0.5 indicate more random, non-correlated, highly constrained behavioural variation (i.e., white noise); *α* values closer to 1.0 indicate moderately persistent, long-range correlated, more flexible, and less constrained behaviour (i.e., pink noise); and *α* values closer to 1.5 indicate highly persistent, overly controlled behaviour (i.e., brown noise)^33,34^.

**Fig. 2:**
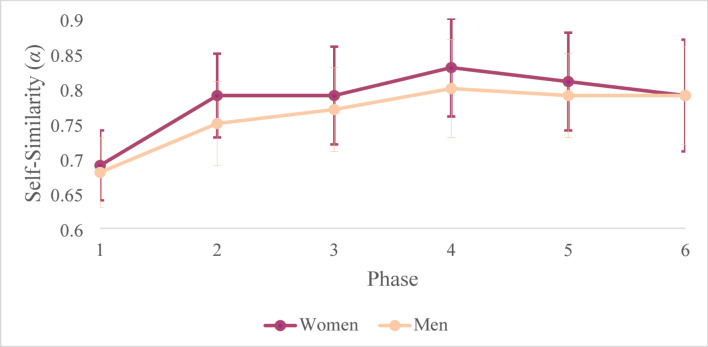
Change in Postural Activity across Phases by Participant Gender. The two-way interaction between phase and participant gender demonstrates differences between women and men over time. Overall, women exhibited higher levels of self-similarity as measured by *α*. However, all participants attained a statistically equivalent state of reduced constraint (*α* closer to 1.0) at end-state compared to baseline (*α* closer to 0.5).

Of particular interest here, was the degree to which participants’ postural activity became more persistent or less constrained throughout the RPG (i.e., within-subjects), and whether these patterns of change differed by participant and colleague gender (i.e., between-subjects). Thus, we employed a 2 (between subjects; participant gender: woman, man) × 2 (between-subjects; colleague gender: transgender, cisgender) × 6 (within-subjects; phase) GLMM with *α* as the dependent variable and an unstructured covariance matrix. A sequential Bonferroni correction was applied to account for multiple comparisons. As described in Table 1, results of the GLMM suggested no significant effects for colleague gender, nor for the two- and three-way interactions involving this factor. However, we observed a two-way interaction between phase and participant gender, as displayed in Fig. 2. Although, *α* increased from baseline (Phase 1) to the conclusion of the RPG (Phase 6), and women exhibited higher values of *α* overall, women were significantly higher than men at Phase 2, Phase 4, and Phase 5.

**Table 1:** GLMM Fixed Effects for the Overall Model.

	*F*	df1	df2	*p*
Corrected Model	32.76	23	217	< .001**
Colleague	0.45	1	148	.502
Gender	11.36	1	148	< .001**
Phase	128.71	5	145	< .001**
Colleague ^x^ Gender	0.03	1	148	.860
Colleague ^x^ Phase	0.95	5	145	.453
Gender ^x^ Phase	2,52	5	145	.032*
Colleague ^x^ Gender ^x^ Phase	1.29	5	145	.270

‘Colleague’ denotes the categorical variable colleague gender (cisgender = 0, transgender = 1), while ‘Gender’ denotes the categorical variable participant gender (women = 0, men = 1). *p < .05. **p < .001.

Additionally, women and men displayed similar patterns of change within-subjects across phases, with two exceptions. From Phase 2 to Phase 3, men’s *α* significantly increased, while women’s α showed no significant change. Conversely, from Phase 5 to Phase 6, women’s *α* significantly decreased, while men’s α showed no significant change. Pairwise comparisons supporting these effects are summarised in Table 2. Taken together, these results indicate that both women and men attain a state of reduced constraint on their behaviour over

**Table 2:** GLMM Pairwise Comparisons by Phase and Participant Gender.

Between-subjects						
Phase	Estimate	*SE*	*t*	df	Lower CI	Upper CI
1	0.01	0.01	1.71	157	−0.01	0.03
2**	0.04	0.01	4.46	156	0.02	0.06
3	0.02	0.01	1.55	153	−0.01	0.03
4*	0.03	0.01	2.82	156	0.01	0.05
5*	0.03	0.01	2.56	157	0.01	0.05
6	0.01	0.01	0.16	122	−0.02	0.02

	Estimate	*SE*	*t*	df	Lower CI	Upper CI
*Within-subjects*						
Women						
1 vs 2**	0.10	0.01	12.28	137	0.08	0.11
2 vs 3	0.01	0.01	0.15	139	−0.02	0.02
3 vs 4**	0.04	0.01	4.44	105	0.02	0.06
4 vs 5	−0.02	0.01	−1.87	128	−0.04	0.01
5 vs 6*	−0.03	0.01	−2.86	102	−0.05	−0.01
Men						
1 vs 2**	0.07	0.01	9.43	151	0.05	0.08
2 vs 3*	0.03	0.01	2.92	150	0.01	0.04
3 vs 4*	0.03	0.01	3.06	185	0.01	0.04
4 vs 5	−0.01	0.01	−1.46	155	−0.03	0.01
5 vs 6	0.01	0.01	0.28	201	−0.01	0.02

A significant two-way interaction between phase and participant gender was observed. Between-subjects effects summarise comparisons of *α* between women and men for each phase. Within-subjects effects summarise the change in *α* between each phase, in series, for women and men. CI = confidence interval. *p < .05. **p < .001.

the course of the interaction (i.e., exhibited more relaxed, less overtly controlled postural sway activity). However, women reach this state earlier in the RPG compared to men, implying that women adapted more readily to the interaction context. Such adaptation reflects behaviour that is not random or reactive from moment to moment^32,34^. Rather, emerging behaviour is shaped by an increasingly stable internal process, in which more coherent patterns of control support smoother and more responsive engagement with the environment or task.

### Colleague and participant gender differentially influence empathy and affiliation

Prior to mediation analyses, we evaluated group means and variances among all self-report measures to describe the fundamental relationships within our data. Differences by participant and colleague gender for independent and repeated measures are presented in Tables 3 and 4, respectively. Regarding between-subjects effects, we observed that women reported significantly higher levels of perceived avatar embodiment as well as higher affective, but not cognitive, trait empathy compared to men. Conversely, men endorsed gender essentialist attitudes and beliefs to a greater extent compared to women. However, social desirability did not differ based on participant or colleague gender.

**Table 3:** Independent Measures Statistics by Colleague and Participant Gender.

	Women Participants		Men Participants		Participant Gender	Colleague Gender	Interaction
	Trans Colleague	Cis Colleague	Trans Colleague	Cis Colleague		*F*_(1, 158)_ (η_p_^2^)	
	*M* (*SD*)						
QCAE-COG	62.15 (5.82)	61.10 (6.97)	59.43 (7.63)	59.85 (6.67)	3.44 (.021)	0.09 (.001)	0.47 (.003)
QCAE-AFF	25.75 (3.84)	25.30 (3.41)	22.86 (3.52)	21.98 (3.72)	29.75** (.158)	1.37 (.009)	0.14 (.001)
MCSDS	20.75 (3.14)	20.93 (3.22)	19.74 (2.56)	20.88 (3.30)	1.22 (.008)	1.86 (.012)	1.00 (.006)
EQ	4.17 (0.55)	4.24 (0.68)	3.88 (0.73)	3.88 (0.78)	8.99* (.054)	0.13 (.001)	0.09 (.001)
HABS-ESG	2.94 (1.45)	2.83 (1.32)	4.05 (1.60)	3.83 (1.44)	21.03** (.117)	0.51 (.003)	0.05 (.000)

QCAE = Questionnaire of Cognitive and Affective Empathy (COG = cognitive empathy dimension; AFF = affective empathy dimension). MCSDS = Marlowe Crowne Social Desirability Scale (social desirability). EQ = Embodiment Questionnaire (virtual embodiment). HABS-ESG = Heteronormative Attitudes and Beliefs Scale–Essential Sex and Gender (gender essentialism). Partial eta squared (η_p_^2^) is reported as a measure of effect size for between-subjects comparisons using two-way ANOVA. *p < .05. **p < .001.

**Table 4:** Mixed-Design ANOVA for Repeated Measures.

Empathy						
Within-subjects	*F*_(3, 474)_	η_p_^2^	EM Means			
Empathy	230.73**	.594		*M*	*SE*	95% CI
Empathy*Gender	1.65	.010	Within			
Empathy*Colleague	0.68	.004	Time 1	5.80	(0.13)	5.55, 6.04
Empathy*Gender*Colleague	0.85	.005	Time 2	5.85	(0.12)	5.61, 6.09
Between-subjects	*F*_(1, 158)_		Time 3	6.57	(0.13)	6.21, 6.73
Gender	19.09**	.108	Time 4	7.76	(0.13)	7.51, 8.01
Colleague	0.08	.001				
Gender*Colleague	0.13	.001	Between			
Pairwise Contrasts	*F*_(1, 158)_		Women	6.97	0.16	6.65, 7.29
1 vs 2	0.67	.004	Men	5.97	0.16	5.65, 6.29
2 vs 3	83.20**	.345				
3 vs 4	198.41**	.557				
*Likeability*						
Within-subjects	*F*_(1, 158)_	η_p_^2^	EM Means			
Likeability	189.61**	.545		*M*	*SE*	95% CI
Likeability*Gender	0.21	.001	Within			
Likeability*Colleague	0.69	.004	Pre	5.61	0.11	5.40, 5.83
Likeability*Gender*Colleague	1.74	.011	Post	7.22	0.13	6.97, 7.48
Between-subjects	*F*_(1, 158)_					
Gender	23.04**	.127	Between			
Colleague	0.67	.004	Women	6.91	0.15	6.62, 7.20
Gender*Colleague	0.48	.003	Men	5.92	0.15	5.63, 6.21
*Similarity*						
Within-subjects	*F*_(1, 158)_	η_p_^2^	EM Means			
Similarity	53.15**	.252		*M*	*SE*	95% CI
Similarity*Gender	0.01	.000	Within			
Similarity*Colleague	49.85**	.240	Trans_(pre)_	3.08	0.19	2.71, 3.46
Similarity*Gender*Colleague	0.02	.000	Cis_(pre)_	5.51	0.19	5.13, 5.89
Between-subjects	*F*_(1, 158)_		Trans_(post)_	5.42	0.22	4.99, 5.86
Gender	5.89*	.036	Cis_(post)_	5.56	0.22	5.11, 5.99
Colleague	27.57**	.149				
Gender*Colleague	0.40	.003	Between			
			Women	5.19	.17	4.85, 5.53
			Men	4.60	.17	4.26, 4.94

Gender = participant gender (women = 0, men = 1). Colleague = colleague gender (cisgender = 0, transgender = 1). State empathy is measured across for timepoints (Time 1, Time 2, Time 3, Time 4), while likeability and similarity are measured pre- and post-RPG. Estimated marginal (EM) means are reported for significant effects within- and between-subjects. Partial eta squared (η_p_^2^) is reported as a measure of effect size. **p < .05. **p < .001.

Regarding repeated measures, empathy toward the colleague was recorded at four timepoints within the RPG, while interpersonal affiliation (i.e., likability, similarity) with the colleague was reported pre- and post-RPG (see Methods for further detail). Using a series of mixed-design ANOVAs, we observed that both empathy and affiliation significantly increased over time, and women provided higher ratings for empathy, likeability, and similarity compared to men. Further examination of the pairwise contrasts revealed that empathy increased from Time 2 to Time 3 (following discussions about workplace equity), and Time 3 to Time 4 (following the debrief), although there was no significant change between Time 1 and Time 2. Despite the presence of these main effects, the two- and three-way interactions involving participant gender were not significant. Similarly, neither state empathy nor likeability differed based on the colleague’s gender. However, the model evaluating similarity demonstrated an effect of colleague gender on perceptions of interpersonal similarity, such that only those who interacted with a transgender colleague reported increased feelings of similarity post-RPG. Specifically, participants reported lower feelings of similarity with a transgender colleague compared to a cisgender colleague before contact, whereas after contact, participants reported equivalent levels of similarity with either colleague.

### Empathy as a pathway from embodiment to gender essentialism and affiliation

To test whether virtual embodiment was associated with post-session gender essentialism and within-session changes in interpersonal affiliation via state empathy, we first examined zero-order correlations among all self-report measures. Given that postural dynamics (indexed via DFA across phases of the simulation) were measured prior to the post-RPG self-reports, we also examined zero-order correlations among these variables (see Table 5). Correlations between virtual embodiment, state empathy, and the outcomes of interest (i.e., gender essentialism, likeability, similarity) indicated suitability for mediation analyses (moderate-to-large correlations). Further, the correlations among state empathy measured across four timepoints supported use of serial mediation (i.e., correlations > 0.70)^35^. Given that preceding results demonstrated differences by participant and colleague gender, we initially used Model 92 from Hayes^35^ PROCESS macro (version 4.2) to assess for moderated mediation in the indirect pathways from embodiment (X) to gender essentialism (Y_1_), likeability (Y_2_), and similarity (Y_3_) via empathy (M_1-4_). We further included trait cognitive and affective empathy as covariates in each model to assess whether context-specific empathy was associated with outcomes over and above one’s dispositional and stable empathetic tendencies. Finally, as social desirability and DFA (Phases 4–6) were correlated with the outcomes of interest, these variables were examined as potential covariates. However, only DFA (Phase 6) remained statistically significant in the model for gender essentialism, and therefore the remaining variables were excluded from final analyses for the sake of model parsimony.

**Table 5:** Zero-order Pearson Correlation Coefficients.

	1	2	3	4	5	6	7	8	9	10	11	12	13	14	15	16	17	18	19
1. QCAE-COG	–																		
2. QCAE-AFF	.08	–																	
3. MCSDS	.12	−.03	–																
4. EQ	.02	.25*	.07	–															
5. HABS-ESG	−.06	−.42*	.10	−.26*	–														
6. Likeability_pre_	.07	.25*	.19*	.22*	−.32*	–													
7. Likeability_post_	.14	.32*	.07	.46*	−.43*	.57*	–												
8. Similarity_pre_	.05	.09	.05	.19*	−.17*	.44*	.39*	–											
9. Similarity_post_	.15	.20*	.08	.37*	−.28*	.39*	.61*	.35*	–										
10. Empathy_1_	.13	.32*	.15	.30*	−.30*	.52*	.62*	.32*	.44*	–									
11. Empathy_2_	.05	.29*	.10	.35*	−.35*	.48*	.60*	.28*	.43*	.87*	–								
12. Empathy_3_	.11	.35*	.16*	.35*	−.37*	.53*	.64*	.29*	.46*	.81*	.87*	–							
13. Empathy_4_	.22*	.40*	.16*	.36*	−.43*	.55*	.75*	.30*	.48*	.72*	.73*	.77*	–						
14. DFA_1_	.01	.04	−.01	.07	−.04	.07	.05	.17*	.02	−.02	−.01	.02	.03	–					
15. DFA_2_	.06	.15	.18*	.08	−.05	.09	.01	.06	.07	.01	−.09	−.07	.02	.23*	–				
16. DFA_3_	−.08	.03	.05	−.03	0.01	.05	.05	.01	.05	.09	.01	.02	.01	.25*	.17*	–			
17. DFA_4_	−.06	.18*	.00	.16*	−.15	.02	.09	.08	−.02	.07	.06	.03	.09	.27*	.28*	.32*	–		
18. DFA_5_	−.05	.22*	−.01	.08	−.05	.11	.09	−.03	−.01	.18*	.18*	.15	.15	.23*	.27*	.32*	.34*	–	
19. DFA_6_	−.03	−.08	−.07	.00	.17*	−.04	−.03	−.01	.02	−.02	−.02	−.04	.03	.22*	.13	.37*	.21*	.36*	–

DFA = detrended fluctuation analysis. *p < .05. Cognitive and affective empathy were included as covariates in all models. Shading denotes correlations between the key predicting, mediating, and outcome variables evaluated. Small, positive, significant correlations with these key variables (underlined, bold) were examined as potential covariates in the mediation models. As neither social desirability nor postural activity at Phases 4 and 5 were significantly associated with the mediators (state empathy) or outcome variables (gender essentialism, likeability_post_, similarity_post_), these variables were excluded from the final mediation models. Postural activity at Phase 6 was retained only in the model assessing gender essentialism.

### Virtual embodiment and gender essentialism via state empathy over time

**Fig. 3:**
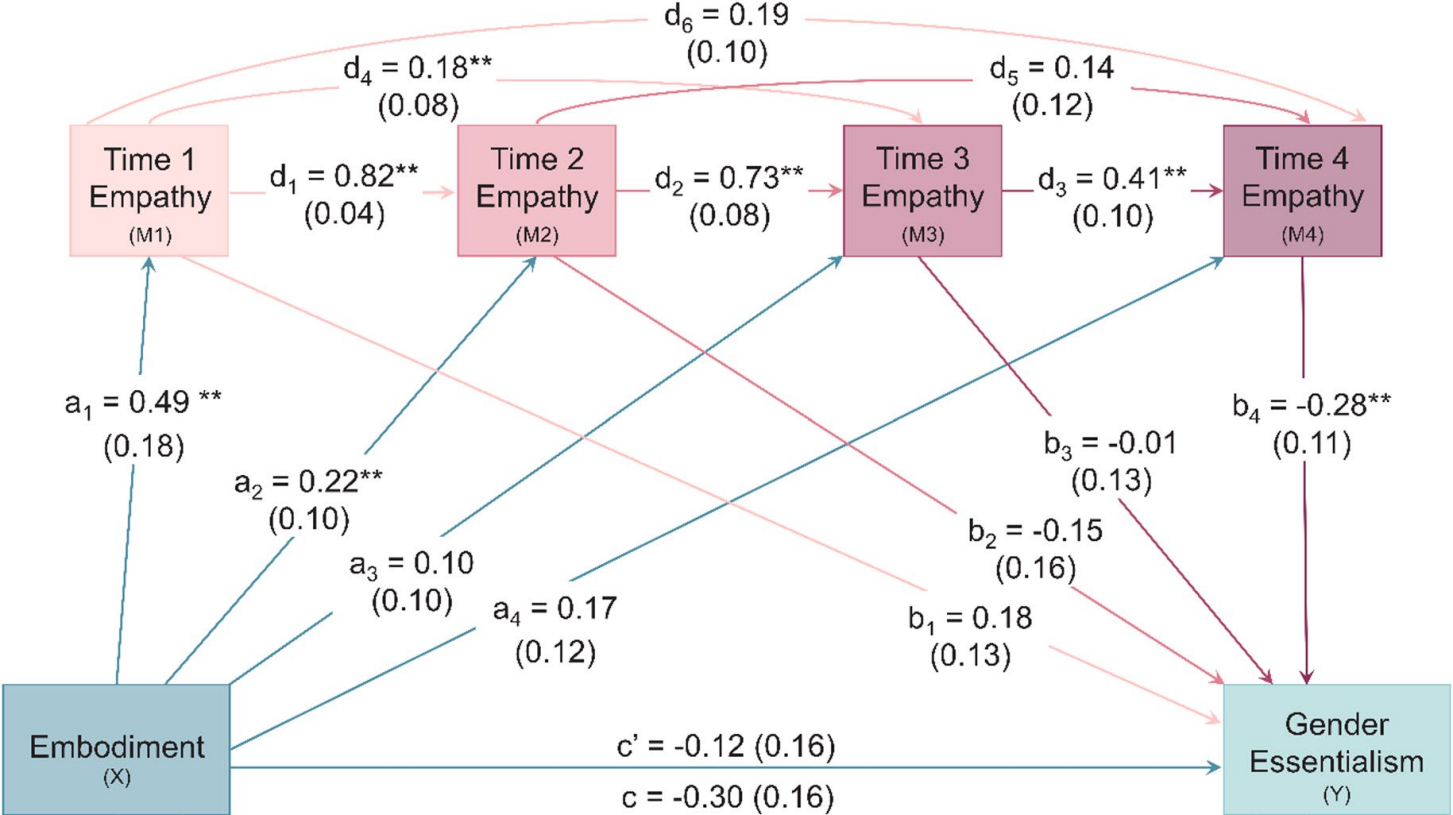
Serial Mediation Model of Gender Essentialism. Increases in empathy over time were consistent with serial mediation for the relationship between virtual embodiment and post-session gender essentialism, with participant and colleague gender, cognitive and affective empathy, and postural activity (*α*) at Phase 6 included as covariates. The total effect is displayed below the horizontal line, while the direct effect is displayed above the line.

Although DFA was not conceptualised as an outcome of the mediation models, exploratory correlations and model testing indicated that end-state DFA (Phase 6) was significantly associated with post-session gender essentialism. Thus, we included this factor as an additional covariate here to assess the robustness of the mediation model to end-state postural dynamics. As analyses with Model 92 suggested no effects consistent with moderated mediation, we used Model 6 for the final analysis while retaining participant and colleague gender as covariates. Figure 3 summarises the serial mediation model where virtual embodiment is negatively associated with endorsement of post-session gender essentialism via state empathy (*F*_(10,151)_ = 7.30, *p* < 0.001, *R*^*2*^ = 0.33).

Despite the previously observed zero-order relationships between virtual embodiment and gender essentialism, neither the direct nor total effects were significant when accounting for covariates and mediators. However, we observed significant indirect effects for the pathway from embodiment to gender essentialism via state empathy at all timepoints, as well as the pathway excluding Time 1 empathy. Given the minimal changes in empathy from Time 1 to Time 2, this latter effect is not overly surprising. Regarding covariates, affective empathy was positively associated with state empathy at Time 1 and negatively associated with gender essentialism, while cognitive empathy was positively associated with state empathy at Time 4. These covariate associations not only indicate that the affective and cognitive dimensions of trait empathy are differentially related to state empathy across the RPG, but also they support the independence of the association between state empathy and gender essentialism when controlling for empathetic disposition. Additionally, end-state DFA (Phase 6) remained negatively associated with gender essentialism, suggesting that end-state postural dynamics explained variance in gender essentialism over and above embodiment and empathy. The indirect effects and covariate statistics supporting these findings are summarised in Table 6. Taken together, these effects suggest complexity in the relationships between embodiment, empathy, and gender essentialism. Neither the sense of embodiment nor empathy alone were associated with the endorsement of gender essentialist attitudes and beliefs after the RPG. Instead, embodiment is first associated with empathy, which in turn increases throughout the RPG, where end-state empathy, in turn, is negatively associated with gender essentialism.

**Table 6:** Indirect Effects and Covariates for Model 6 Predicting Gender Essentialism.

Indirect Effect	Coefficient (*SE*)	95% CI	Effect Size (*SE*)	95% CI
Total*	−0.18 (0.08)	−0.35, −0.04	−0.08 (0.04)	−0.16, −0.12
a_1_b_1_	0.09 (0.07)	−0.03, 0.26	0.04 (0.03)	−0.01, 0.12
a_2_b_2_	−0.03 (0.04)	−0.12, 0.03	−0.02 (0.02)	−0.06, 0.01
a_3_b_3_	−0.01 (0.02)	−0.04, 0.03	−0.01 (0.01)	−0.02, 0.01
a_4_b_4_	−0.05 (0.05)	−0.16, 0.03	−0.02 (0.02)	−0.07, 0.01
a_1_d_1_b_2_	−0.06 (0.07)	−0.21, 0.07	−0.03 (0.03)	−0.10, 0.03
a_1_d_4_b_3_	−0.01 (0.01)	−0.03, 0.03	−0.01 (0.01)	−0.01, 0.02
a_1_d_6_b_4_	−0.03 (0.02)	−0.08, 0.00	−0.01 (0.01)	−0.04, 0.00
a_2_d_2_b_3_	−0.01 (0.02)	−0.05, 0.05	−0.01 (0.01)	−0.02, 0.02
a_2_d_5_b_4_	−0.01 (0.01)	−0.03, 0.01	−0.01 (0.01)	−0.01, 0.01
a_3_d_3_b_4_	−0.01 (0.01)	−0.04, 0.01	−0.01 (0.01)	−0.02, 0.01
a_1_d_1_d_2_b_3_	−0.01 (0.04)	−0.08, 0.08	−0.01 (0.02)	−0.04, 0.04
a_1_d_1_d_5_b_4_	−0.02 (0.02)	−0.05, 0.02	−0.01 (0.01)	−0.03, 0.01
a_1_d_4_d_3_b_4_	−0.01 (0.01)	−0.04, 0.01	−0.01 (0.01)	−0.02, 0.01
a_2_d_2_d_3_b_4_*	−0.02 (0.02)	−0.06, −0.01	−0.01 (0.01)	−0.03, −0.01
a_1_d_1_d_2_d_3_b_4_*	−0.03 (0.03)	−0.10, −0.01	−0.02 (0.01)	−0.05, −0.01

Covariates	Coefficient	*SE*	Lower CI	Upper CI
*Time 1 Empathy (M1)*				
Cognitive	0.02	0.02	−0.02	0.06
Affective*	0.08	0.03	0.02	0.15
Gender	−0.48	0.27	−1.01	0.04
Colleague	0.03	0.24	−0.44	0.51
End-state DFA	0.01	1.64	−3.22	3.25
*Time 2 Empathy (M2)*				
Cognitive	−0.01	0.01	−0.03	0.01
Affective	0.01	0.02	−0.04	0.04
Gender	−0.02	0.14	−0.30	0.27
Colleague	−0.11	0.13	−0.37	0.14
End −state DFA	−0.12	0.88	−1.85	1.61
*Time 3 Empathy (M3)*				
Cognitive	0.01	0.01	−0.01	0.03
Affective	0.03	0.02	−0.01	0.07
Gender	−0.08	0.15	−0.37	0.21
Colleague	0.10	0.13	−0.16	0.36
End-state DFA	−0.42	0.90	−2.20	1.36
*Time 4 Empathy (M4)*				
Cognitive*	0.03	0.01	0.01	0.06
Affective	0.05	0.02	−0.01	0.09
Gender	−0.25	0.18	−0.60	0.10
Colleague	0.09	0.16	−0.22	0.41
End-state DFA	1.63	1.09	−0.51	3.78
Gender Essentialism (Y)				
Cognitive	0.01	0.02	−0.02	0.04
Affective*	−0.09	0.03	−0.15	−0.03
Gender	0.44	0.23	−0.03	0.89
Colleague	0.23	0.21	−0.18	0.64
End-state DFA*	3.39	1.43	0.58	6.21

*p < .05. Completely standardised indirect effects are reported as a measure of effect size. CI = confidence interval. Gender: 0 = women, 1 = men. Colleague: 0 = cisgender, 1 = transgender.

### Virtual embodiment and interpersonal affiliation with the transgender colleague

**Fig. 4:**
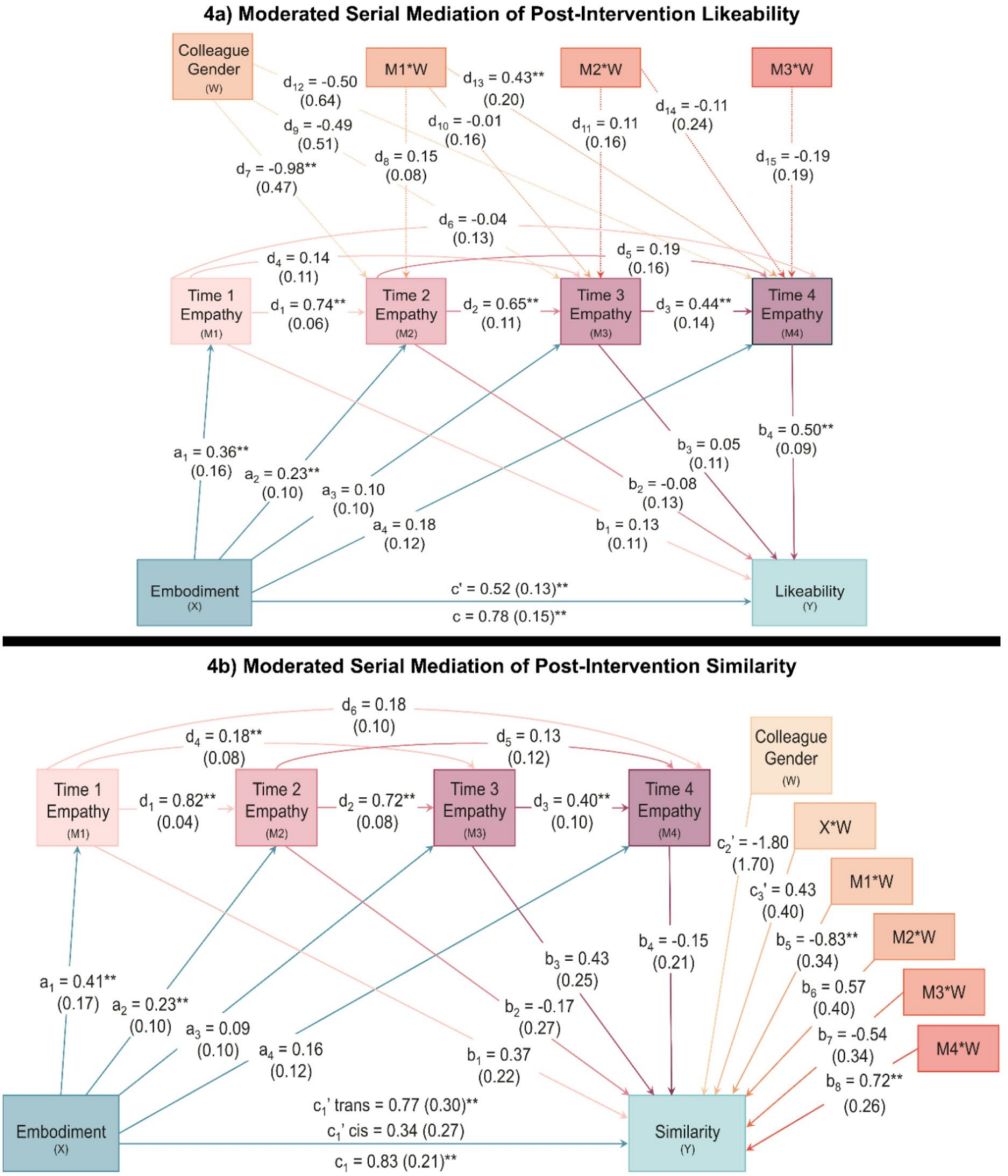
(**a**) Increases in empathy over time were consistent with mediating the relationship between virtual embodiment and likeability ratings following the RPG, but only for those who interacted with the transgender colleague (moderated d-path). Participant gender, cognitive and affective empathy, and baseline likeability were included as covariates. The total effect is displayed below the horizontal line, while the direct effect is displayed above the line. (**b**) Increases in empathy over time were consistent with mediating the relationship between virtual embodiment and similarity ratings following the RPG, but only for those who interacted with the transgender colleague (moderated b-path) Participant gender, cognitive and affective empathy, and baseline similarity were included as covariates. The total effect is displayed below the horizontal line, while the conditional (moderated) direct effects are displayed above the line for the transgender and cisgender colleagues.

Figure 4a summarises the moderated serial mediation model for perceived likeability toward the colleague at post-RPG, holding constant perceived likeability at baseline (*F*_(9, 152)_ = 30.14, *p* < 0.001, *R*^*2*^ = 0.64). Our exploratory analyses using Model 92 suggested that colleague gender served to moderate only the serial mediators. Thus, Model 91 was used for the final analysis, retaining participant gender as a covariate. Here, a significant indirect effect was observed only for those who interacted with the transgender colleague, such that the relationship between embodiment and likeability was consistent with mediation for Time 1 followed by Time 4 empathy. As illustrated in Fig. 4a, the interaction between colleague gender and state empathy at Time 1 was significantly associated with state empathy at Time 4. However, this effect was significant for the transgender (*b* = 0.39, *SE* = 0.15, *p* = 0.012, 95% CI [0.09, 0.69]), but not the cisgender interaction partner (*b* =  −0.04, *SE* = 0.13, *p* = 0.765, 95% CI [ −0.29, 0.22]). In other words, the pattern consistent with moderated mediation observed here suggests that although all participants reported increased feelings of likeability toward their interaction partner following the RPG simulation, it is the increase from baseline to end-state empathy that appears to matter for more positive attitudes toward a transgender outgroup target.

The full indirect effects and covariates table for this model can be viewed in Table 7. Similar to the previous model, affective empathy was positively associated with state empathy at Time 1, while both affective and cognitive empathy were positively associated with state empathy at Time 4. Thus, we again see support for the differential relationship of both trait and state empathy across the progression of the RPG. Additionally, baseline likeability ratings were associated with post-RPG likeability and empathy at all timepoints except Time 2, where instead, empathy toward the cisgender colleague was significantly higher. This pattern might suggest that one’s initial perceptions of another—at least in terms of favourability, pleasantness, or agreeability—could be meaningfully related to how empathy develops across the course of an interaction. Unlike the previous model, however, the total and direct effects of embodiment on likeability were significant, indicating that embodiment remained associated with likeability when holding constant only the covariates, as well as all mediators and covariates, respectively.

**Table 7:** Indirect Effects and Covariates for Model 91 Predicting Likeability.

Unconditional Indirect Effects				
	*b*	*SE*	Lower CI	Upper CI
a_1_b_1_	0.05	0.05	−0.03	0.16
a_2_b_2_	−0.02	0.03	−0.09	0.04
a_3_b_3_	0.01	0.01	−0.02	0.04
a_4_b_4_	0.09	0.08	−0.04	0.27

	Cisgender Colleague		Transgender Colleague		Index	
	*b* (*SE*)	95% CI	*b* (*SE*)	95% CI	*b* (*SE*)	95% CI
*Conditional Indirect Effects*						
a_1_d_1_b_2_	−0.02 (0.04)	−0.12, 0.05	−0.03 (0.05)	−0.13, 0.06	−0.01 (0.01)	−0.02, 0.01
a_1_d_4_b_3_	0.01 (0.01)	−0.01, 0.03	0.01 (0.01)	−0.01, 0.02	−0.01 (0.01)	−0.03, 0.01
a_1_d_6_b_4_	−0.01 (0.02)	−0.06, 0.03	0.07 (0.05)	0.01, 0.20*	0.08 (0.06)	0.01, 0.22*
a_2_d_2_b_3_	0.01 (0.02)	−0.03, 0.05	0.01 (0.02)	−0.03, 0.06	0.01 (0.01)	−0.01, 0.02
a_2_d_5_b_4_	0.02 (0.02)	−0.01, 0.07	0.01 (0.03)	−0.05, 0.07	−0.01 (0.03)	−0.09, 0.05
a_3_d_3_b_4_	0.02 (0.03)	−0.02, 0.08	0.01 (0.02)	−0.02, 0.05	−0.01 (0.02)	−0.06, 0.02
a_1_d_1_d_2_b_3_	0.01 (0.02)	−0.03, 0.06	0.01 (0.03)	−0.04, 0.08	0.01 (0.01)	−0.01, 0.03
a_1_d_1_d_5_b_4_	0.03 (0.03)	−0.02, 0.09	0.01 (0.04)	−0.08, 0.10	−0.01 (0.05)	−0.12, 0.07
a_1_d_4_d_3_b_4_	0.01 (0.02)	−0.01, 0.05	0.01 (0.01)	−0.01, 0.03	−0.01 (0.02)	−0.05, 0.03
a_2_d_2_d_3_b_4_	0.03 (0.03)	−0.01, 0.10	0.02 (0.02)	−0.01, 0.07	−0.01 (0.03)	−0.08, 0.03
a_1_d_1_d_2_d_3_b_4_	0.04 (0.03)	−0.01, 0.11	0.03 (0.03)	−0.01, 0.10	−0.01 (0.03)	−0.08, 0.05

Covariates	*b*	*SE*	Lower CI	Upper CI
*Time 1 Empathy (M1)*				
Cognitive	0.02	0.02	−0.01	0.05
Affective*	0.07	0.03	0.01	0.13
Gender	−0.08	0.25	−0.56	0.41
Likeability (pre)*	0.48	0.08	0.33	0.64
*Time 2 Empathy (M2)*				
Cognitive	−0.01	0.01	−0.03	0.01
Affective	0.01	0.02	−0.03	0.04
Gender	0.01	0.15	−0.28	0.30
Likeability (pre)	0.02	0.05	−0.08	0.13
*Time 3 Empathy (M3)*				
Cognitive	0.01	0.01	−0.01	0.03
Affective	0.03	0.02	−0.01	0.07
Gender	−0.01	0.15	−0.30	0.29
Likeability (pre)*	0.13	0.05	0.03	0.24
*Time 4 Empathy (M4)*				
Cognitive*	0.03	0.01	0.01	0.06
Affective*	0.05	0.02	0.01	0.10
Gender	−0.10	0.18	−0.45	0.26
Likeability (pre)*	0.18	0.07	0.05	0.31
*Likeability (Y)*				
Cognitive	−0.01	0.01	−0.03	0.03
Affective	0.01	0.02	−0.05	0.05
Gender	0.18	0.19	−0.19	0.56
Likeability (pre)*	0.24	0.07	0.10	0.38

*p < .05. A statistically significant index of moderated mediation suggests that the indirect effect is conditional on the values of the moderator. CI = confidence interval. Gender: 0 = women, 1 = men. Pre = baseline likeability.

Figure 4b displays the moderated mediation model for perceived similarity with the colleague at post-RPG, holding constant perceived similarity at baseline (*F*_(15, 146)_ = 6.57, *p* < 0.001, *R*^*2*^ = 0.40). Here, Model 92 suggested that colleague gender served to moderate only the b-path. Thus, we used Model 89 for the final analysis, retaining participant gender as a covariate. Reflecting our earlier mixed-design ANOVA, the direct effect was significant only for those who interacted with the transgender colleague. Likewise, a significant indirect effect was observed only for the transgender colleague, such that embodiment was associated with increased similarity via state empathy across all timepoints. Figure 4b denotes two significant interactions between colleague gender and empathy at Time 1 and Time 4. An examination of the conditional simple effects revealed that the strength of the relationship between Time 1 empathy and similarity did not significantly differ between transgender (*b* =  −0.46, *SE* = 0.27, *p* = 0.086, 95% CI [ −0.99, 0.07]) and cisgender colleagues (*b* = 0.37, *SE* = 0.22, *p* = 0.094, 95% CI [ −0.06, 0.80]), although the direction of these effects were in opposition to each other.

However, the strength of the relationship between Time 4 empathy and similarity was significant only for the transgender ((*b* = 0.57, *SE* = 0.17, *p* = 0.001, 95% CI [0.23, 0.91]), and not the cisgender colleague (*b* =  −0.15, *SE* = 0.21, *p* = 0.470, 95% CI [ −0.56, 0.26]). The pattern consistent with moderated mediation observed here again suggests that end-state empathy is important for more positive attitudes, in particular for greater feelings of closeness and lower psychological distance with those who are initially perceived as dissimilar or ‘other’. Further aligning with the previous models, affective empathy was positively associated with state empathy at Time 1, while cognitive empathy was positively associated with state empathy at Time 4. However, baseline similarity ratings were only associated with empathy at Time 1 and similarity at post-RPG, indicating a more limited role of one’s initial interpersonal perceptions. Table 8 summarises the indirect effects and covariates for this model.

**Table 8:** Indirect Effects and Covariates for Model 89 Predicting Similarity.

	Cisgender Colleague		Transgender Colleague		Index	
Effect	*b* (*SE*)	95% CI	*b* (*SE*)	95% CI	*b* (*SE*)	95% CI
a_1_b_1_	0.15 (0.12)	−0.07, 0.41	−0.19 (0.16)	−0.55, 0.07	−0.34 (0.22)	−0.81, 0.02
a_2_b_2_	−0.04 (0.09)	−0.27, 0.10	0.09 (0.10)	−0.06, 0.32	0.13 (0.14)	−0.06, 0.49
a_3_b_3_	0.04 (0.06)	−0.06, 0.19	−0.01 (0.03)	−0.08, 0.06	−0.05 (0.07)	−0.22, 0.08
a_4_b_4_	−0.02 (0.08)	−0.26, 0.06	0.09 (0.09)	−0.07, 0.27	0.12 (0.14)	−0.84, 0.46
a_1_d_1_b_2_	−0.06 (0.12)	−0.29, 0.20	0.13 (0.14)	−0.10, 0.46	0.19 (0.18)	−0.14, 0.57
a_1_d_4_b_3_	0.03 (0.04)	−0.01, 0.13	−0.01 (0.02)	−0.06, 0.04	−0.04 (0.05)	−0.16, 0.02
a_1_d_6_b_4_	−0.01 (0.03)	−0.09, 0.03	0.04 (0.04)	−0.01, 0.13	0.05 (0.05)	−0.01, 0.18
a_2_d_2_b_3_	0.07 (0.08)	−0.01, 0.28	−0.02 (0.05)	−0.12, 0.08	−0.09 (0.10)	−0.35, 0.02
a_2_d_5_b_4_	−0.01 (0.01)	−0.04, 0.02	0.02 (0.03)	−0.01, 0.09	0.02 (0.03)	−0.02, 0.10
a_3_d_3_b_4_	−0.01 (0.02)	−0.05, 0.03	0.02 (0.03)	−0.02, 0.08	0.03 (0.04)	−0.04, 0.11
a_1_d_1_d_2_b_3_	0.11 (0.09)	−0.01, 0.35	−0.03 (0.07)	−0.18, 0.12	−0.13 (0.12)	−0.44, 0.04
a_1_d_1_d_5_b_4_	−0.01 (0.02)	−0.05, 0.03	0.03 (0.04)	−0.02, 0.12	0.03 (0.04)	−0.04, 0.13
a_1_d_4_d_3_b_4_	−0.01 (0.01)	−0.05, 0.01	0.02 (0.02)	−0.01, 0.06	0.02 (0.03)	−0.01, 0.09
a_2_d_2_d_3_b_4_	−0.01 (0.03)	−0.10, 0.02	0.04 (0.03)	−0.01, 0.11	0.05 (0.05)	−0.01, 0.19
a_1_d_1_d_2_d_3_b_4_	−0.02 (0.04)	−0.14, 0.03	0.06 (0.04)	0.01, 0.15*	0.07 (0.06)	0.01, 0.24*

Covariates	*b*	*SE*	Lower CI	Upper CI
*Time 1 Empathy (M1)*				
Cognitive	0.02	0.02	−0.02	0.05
Affective*	0.08	0.03	0.01	0.14
Gender	−0.41	0.26	−0.92	0.09
Similarity (pre)	0.18	0.06	0.07	0.29
*Time 2 Empathy (M2)*				
Cognitive	−0.02	0.01	−0.03	0.01
Affective	0.01	0.02	−0.04	0.04
Gender	−0.02	0.14	−0.30	0.26
Similarity (pre)	−0.05	0.03	−0.07	0.06
*Time 3 Empathy (M3)*				
Cognitive	0.01	0.01	−0.01	0.03
Affective	0.03	0.02	−0.01	0.07
Gender	−0.07	0.15	−0.36	0.22
Similarity (pre)	0.03	0.03	−0.04	0.09
*Time 4 Empathy (M4)*				
Cognitive*	0.03	0.01	0.01	0.06
Affective	0.04	0.02	−0.01	0.09
Gender	−0.25	0.18	−0.60	0.10
Similarity (pre)	0.03	0.04	−0.05	0.11
*Similarity (Y)*				
Cognitive	0.03	0.02	−0.01	0.07
Affective	−0.03	0.04	−0.10	0.05
Gender	0.24	0.29	−0.34	0.82
Similarity (pre)*	0.28	0.09	0.11	0.45

*p < .05. A statistically significant index of moderated mediation suggests that the indirect effect is conditional on the values of the moderator. CI = confidence interval. Gender: 0 = women, 1 = men. Pre = baseline similarity.

## Discussion

In this study, we evaluated a virtual RPG designed to support cisgender-transgender interactions and address transprejudice. Our findings suggest that state empathy may play a unique role in shaping attitudinal responses among cisgender majority group members following embodied interactions in VR. These findings advance recent work that has demonstrated the potential of VRPT and VR-mediated contact as independent approaches to prejudice reduction in other identity domains^7,8^ by employing both approaches simultaneously and providing preliminary evidence for their impact on gender essentialist beliefs and cisgender-transgender affiliation. By capturing non-verbal postural dynamics across the interaction, we offer novel insight into how embodied processes shift across key perturbations of the VR interaction.

Extending previous work on binary gender^17^, we found evidence of an indirect association between perceived virtual embodiment and post-session gender essentialism via state empathy for all participants, such that higher embodiment was associated with greater empathy, which in turn was associated with lower endorsement of gender essentialism. We also observed a novel pattern for within-session affiliation, whereby virtual embodiment was associated with increased empathy, which in turn was associated with increased feelings of likeability and similarity, but only among those who interacted with a transgender colleague. Taken together, these findings suggest that VRPT (e.g., embodying a transgender avatar) may be a promising approach for engaging with internalised cognitions about gendered norms that precede transprejudice and discrimination^36^. However, it should be noted that our mediation analyses tested conditional associations consistent with, but not wholly demonstrating, the proposed empathy-based mechanism linking embodiment to essentialism and affiliation. To further evaluate this initial result, future work should experimentally manipulate virtual embodiment (e.g., comparing transgender and cisgender embodiment) to determine whether induced differences in embodiment produce corresponding changes in state empathy and downstream shifts in essentialist beliefs or intergroup affiliation.

Alongside VRPT, VR-mediated contact may offer an additional benefit for facilitating opportunities to develop a sense of shared identity. As described above, we observed within-session increases in likeability and similarity only when cisgender individuals both embody a transgender outgroup identity and interact with a transgender outgroup member. Thus, our findings suggest that combining perspective taking and contact within VR contexts may be a promising direction for future prejudice reduction research. In addressing transprejudice, the roles of likeability and similarity should not be overlooked. Fostering recognition of shared similarities can reduce anxiety about and avoidance of those who deviate from traditional, binary gender norms^5^. Moreover, enhancing these factors contributes to increased readiness for future intergroup interactions^37^, counteracting pervasive negative stereotypes that construe transgender individuals as ‘other’. However, it should be noted that within-session affiliation was measured via single likeability and similarity items in the current study. To extend this initial finding, future research could evaluate the multidimensional nature of intergroup perceptions using more comprehensive measures of interpersonal affiliation, such as multi-item scales^38^, semantic differential ratings^39^, or social distance metrics^40^.

Using non-linear fractal analysis (i.e., DFA) to examine postural activity dynamics, we also found that undertaking the RPG simulation allowed participants to achieve a state of reduced constraint on their behaviour. In other words, the increased complexity of participants’ postural behaviours indicated an emerging pattern toward a balanced state of predictability and variability, in which past behaviour has a measurable influence on future behaviour^41^. These behavioural states are characterised by adaptability and resilience in response to internal disruptions or environmental changes^26,27^, such as those inherent in VRPT (e.g., embodied cognition, simulated context, virtual realism). While the colleague’s gender identity did not appear to influence postural activity, we observed that women achieved a more adaptable and resilient state earlier in the RPG than men. However, men exhibited a significant increase in complexity following discussions that specifically focused on allyship, gender identity, and equity in the workplace. Given that women are often subject to persistent experiences of prejudice and discrimination at work^42^, they may shift more readily into the VRPT experience of embodying a transgender identity. Aligning with previous work^43,44^, men may require exposure to specific content aimed at highlighting the impact of privilege on marginalised groups before they begin to benefit from the VRPT experience.

These non-verbal behavioural dynamics appear to co-vary with self-reported measures of empathy. First, empathy peaked at the conclusion of the simulation, and both empathy and DFA at this end-state uniquely contributed to variance in post-session gender essentialism. Second, women reported greater empathy than men at all timepoints and exhibited earlier increases in the persistence and flexibility of their postural activity. Third, men exhibited increases in postural complexity following discussions regarding workplace allyship, gender identity, and equity, which coincided with a significant increase in state empathy at Time 3. By triangulating postural activity data with self-reported, context-specific empathy, we offer an initial descriptive account of how the affective and cognitive processes implicated in theoretical models of prejudice reduction may fluctuate during simulated interactions. Indeed, previous work has identified empathy as a significant contributor to positive outcomes following VRPT^24^, and as a mediator of intergroup contact and prejudice reduction more broadly^23^. Future work could experimentally manipulate empathetic engagement to test whether the coupling between state empathy and non-verbal postural dynamics reflects a functional relationship.

Extending previous work using VRPT and VR-mediated contact in other intergroup contexts^7,8^, the current study offers two key considerations for the development of future interventions targeting transprejudice. First, simulated interactions should aim to be synchronous, rather than asynchronous (e.g., imagined)^10^ or observational (e.g., parasocial media representations)^11^. While these latter approaches are often preferred for feasibility, their reduced social presence can, in turn, impact interaction quality, interpersonal trust, and outgroup favourability^45^. However, immersive VR interactions remain parasocial in nature, as participants engage with virtual avatars and other computer-generated entities. Accordingly, the present findings should not be interpreted as directly equivalent to real-world, human–human interaction, which involves features such as reciprocal spontaneity, emotional complexity, and genuine unpredictability^46,47^. Rather, VR may represent a higher social presence form of parasocial interaction that more closely approximates certain features of direct contact (e.g., synchrony, contingency, embodied interaction)^48^. Thus, a key direction for future research is to systematically compare human–human and VR-mediated interactions to determine whether and under what conditions observed effects may have practical significance beyond simulated contexts.

Second, simulated cisgender-transgender interactions should aim to enable rapport building, rather than simply exposing majority group individuals to marginalised identities to enhance familiarity. While rapport and familiarity are related, rapport involves a higher degree of emotional connection or interpersonal closeness^49^. Indeed, feelings of closeness with outgroup members are stronger predictors of reduced prejudice and increased acceptance^50^, with efforts to foster such relationships being most effective when they involve personal and meaningful connections^51^. Although our results highlight the potential utility of simulated exposures to gender identity microaggressions (e.g., bathroom access), it is critical for intervention designers to engage in community consultation when representing other aspects of transgender lived experience (e.g., navigating gender-exclusive spaces, accessing medical care, securing workplace or community benefits) in future interventions targeting cisgender individuals.

As a tool for research, VR balances experimental control with ecological validity, more closely approximating direct contact and enabling visceral perspective-taking without relying on the labour of vulnerable populations. However, there are limitations to this work that must be considered. First, certain methodological constraints should be noted. As virtual embodiment was not experimentally manipulated, the serial mediation models are observational with respect to this variable. Additionally, the current study did not include a cisgender avatar embodiment condition or a non-intervention control group, and gender essentialism was assessed only at post-RPG. Accordingly, effects attributable to intervention cannot be definitively established. Certain self-report constructs were also assessed using single-items indicators (i.e., likeability, similarity), and internal consistency values for virtual embodiment, social desirability, and affective trait empathy fell below conventional thresholds (α < 0.70), which may have attenuated or inflated the observed associations. We encourage future research to extend the lines of enquiry initiated in this study by pursuing the trajectories outlined in the preceding discussion and developing extended (e.g., fully crossed) designs that allow for robust tests of intervention effects.

Second, our data was collected among an Australian sample, which may not be representative of individuals on a global scale. Although the Australian context aligns with cultural, legal, and social tensions surrounding gender identity in Western and Global North locations, future work should continue to investigate how interventions can be applied in contexts where transgender identities are criminalised^52,53^ and in conflict or post-conflict settings^54,55^. Third, we did not investigate the impact of multiple exposures. While research suggests that initial exposures may be the strongest predictors of future intentions^37,56^, significantly more longitudinal research is needed to understand the degree to which benefits are sustained in the long term. Finally, participants interacted with avatars that were designed from Asian and White models, created to represent a majority of the Australian university population. Thus, participants were not explicitly matched with an interaction partner based on their own racial or ethnic identities. While the current study targeted gender identity, holding additional marginalised identities may further impact experiences of perspective taking and intergroup contact^57^. As interventions frequently address identity categories independently, this highlights a need for future work that targets intersectional identities, both in VR and in the intervention space more broadly, to better represent the dynamics of real-world diversity.

## Methods

### Participants

Cisgender undergraduates (*N* = 162; Women = 80, Men = 82; *M*_age_ = 20.28, *SD* = 3.17) were recruited from the university’s research participant pool and randomly assigned to interact with either the transgender (Women = 40, Men = 42) or cisgender colleague (Women = 40, Men = 40). A priori power analyses employed a moderate effect size and suggested that 136 participants were needed to achieve 80% power for mixed models with one within- and two between-subjects factors (G*Power), while a range of 150–155 participants was needed for models with four serial mediators (Monte Carlo simulation). We initially recruited 173 participants, although 11 were excluded from the analyses due to incomplete data (e.g., inattention, data recording error, technical difficulties). The final sample was comprised largely of Asian (17.9%) and White (34%) participants, followed by European (16.7%), Middle Eastern (8.6%), Indian (8.6%), Black (1.2%), and Hispanic (1.9%) participants, while 11.1% of participants reported mixed backgrounds. Previous experience with virtual reality was reported by 58% of participants (Limited: *n* = 72, Moderate: *n* = 18; Frequent: *n* = 4). No other demographic data were collected (e.g., religious beliefs, political affiliation), as the current study was not designed to examine individual differences in these variables, although future work may wish to consider their potential moderating role.

### Procedure

In exchange for course credit, undergraduate students volunteered to complete an RPG simulation of an after-work team-building event undertaken within VR. Participants were required to be at least 18 years of age, with normal or corrected-to-normal vision, and no history of motion sickness or neurological, neuromuscular, or skeletal disorders. On arrival at the lab, participants provided voluntary, written, informed consent to undertake the following procedures, as approved by the Macquarie University Human Research Ethics Committee (Project ID: 10,903) in accordance with the requirements set out in the National Statement on Ethical Conduct in Human Research 2007.

First, participants customised one cisgender and one transgender avatar to represent themselves in VR. Guided by the experimenter, participants were allotted 15 min to create each avatar (i.e., 30 min total; see Participant Avatars for further detail). Although we initially informed participants that they would be randomly assigned to embody one of their customised avatars in VR, all participants were required to embody their transgender avatar to enable VRPT. Instead, they were randomly assigned to interact with either a transgender or cisgender virtual colleague (Fig. 1a), such that women were paired with either a cisgender woman or transgender man, whereas men were paired with a cisgender man or transgender woman. The purpose of this deception was to minimise demand characteristics by obscuring the study’s focus on transgender embodiment. Following avatar customisation, participants were presented with information about their assigned colleague (e.g., work history, pronouns, gender identity; see Supplementary Material A) before providing baseline ratings of perceived interpersonal affiliation. Notably, the colleague’s work history indicated that they worked in the information technology field. Replicating previous work^17^, this setting was intentionally selected to situate the simulation within a STEM context, where gender disparities in representation and workplace climate are well-documented^58,59^, making it a relevant backdrop for the present investigation.

Prior to entering VR, participants viewed an instructional video outlining directives for VR gameplay, teleportation within the virtual environment, and how to interact with their colleague using the dialogue engine. The instructional video can be viewed at https://youtu.be/HJRGRy53EsA. Participants then entered VR using the HTC Vive system, where they first appeared in the lobby of an arcade-style gaming centre facing a virtual mirror, in which they could see the reflection of their customised transgender avatar. Here, the experimenter verbally confirmed and reinforced participants’ transgender identity and initiated a movement integration exercise prior to beginning the RPG simulation (see Supplementary Material B).

The RPG then began with participants being introduced to their virtual colleague and practicing navigation via teleportation. Participants progressed through the RPG by playing three games with their colleague, which took place between scripted dialogues designed to build rapport with the virtual colleague, gradually increasing in intimacy (see Dialogue Engine and Scripting for further detail). The first and third games involved competitive (i.e., air hockey) and cooperative tasks (i.e., sheep herding), with presentation of task type counterbalanced across participants, while the second game served to maintain engagement and progress the RPG (i.e., whack-a-mole, neither competitive nor cooperative). Following the completion of gameplay, participants were guided to a virtual bar where the final scripted event sequence commenced, which was designed to serve as an exposure to real-world prejudice and discrimination commonly faced by transgender individuals. First, a drink was spilled, and participants were instructed to wash their hands in the bathroom. Next, participants were required to decide which of two bathrooms to enter (i.e., women’s, men’s). Once inside, they triggered a faucet using their controller to simulate washing their hands, while viewing themselves in a virtual bathroom mirror. Upon exiting, participants encountered another patron in the arcade who challenged their use of the ‘wrong’ bathroom. After the confrontation, participants returned to their virtual colleague, who engaged them in a debrief about the incident and concluded the RPG simulation. Empathy toward the virtual colleague was recorded at four timepoints during the simulation: after the first, second, and third games, as well as following the final debrief. Participants’ postural activity was recorded throughout the RPG (see Data Processing for further detail).

After exiting VR, participants completed a battery of self-report questionnaires, first completing state measures of interpersonal affiliation, perceived sense of embodiment, and gender essentialism, followed by trait measures of empathy and social desirability. Participants were then debriefed about the true purpose of study (i.e., VRPT, VR-mediated contact) and the deception used (i.e., transgender avatar embodiment, random assignment to colleague).

### Materials

#### Apparatus

Participants used the HTC Vive Pro Eye system to view and interact within the virtual environment. This system is comprised of a VR headset with dual-OLED display and a combined resolution of 2880 × 1600 pixels and 615 PPI, two hand-controllers, and wall-mounted base stations. Three Vive 2.0 base stations were affixed to the uppermost portion of the wall to maximise coverage and minimise obstructions. These base stations consistently track and determine the precise position and orientation of the Vive headset and controllers, allowing for real-time motion tracking and avatar motor control.

#### Participant avatars

Participants customised their avatars using Adobe Fuse (version 1.3). To do this, participants first selected their preferred body type from a selection of premade, height-scaled, feminine and masculine models. The model that best matched the participant’s height, within a range of 153–182 cm, was then imported into Adobe Fuse, where sliders were available to adjust size and musculature of the body (e.g., arms, legs, torso, hips, breasts), facial structure (e.g., cheek bones, eye shape, lips, jawline), and features such as skin tone and eye colour. After finalising adjustments to the body, participants were guided through options to choose their avatar’s dynamic features (e.g., clothing, hair style, makeup, facial hair). The same baseline model was used for customisation of both the transgender and cisgender avatars. The customised transgender avatar was then rigged for animation using ActorCore AccuRIG (version 1.2.1) before being imported into the virtual environment. A selection of participant avatars is displayed in Supplementary Material C.

#### Pre-programmed avatars

Figure 1a displays the transgender and cisgender colleague avatars, while the two cisgender aggressor avatars can be viewed in Supplementary Material C. Cisgender avatars were selected from a larger pool of models created by the research team for use in human–computer interaction research, while transgender avatars were specifically designed to minimise stereotypical representations of transgender identities. These avatars were created using Adobe Fuse and Blender 3D modelling software and rigged for animation using Mixamo (https://www.mixamo.com). Avatars were pre-programmed into the virtual environment and synced with pre-recorded voiceover, conducted by transgender and cisgender voice actors, which was integrated with SALSA LipSync software to enable facial animations using an approximation technique.

#### Virtual environment

The virtual environment was created and hosted in Unity (version 2020.3.42f1). Participants first arrived in the lobby of the arcade-style gaming centre, where they viewed their avatar in a virtual mirror. Once the RPG was initiated, this mirror was removed, revealing the full arcade environment. Relative to the participant’s initial position, a welcome desk was located on the right side of the lobby. The air hockey and herding game tables were located at the centre of the gaming area, while whack-a-mole and additional arcade machines lined each side of the room. The bar and dining area was located at the far right, adjacent to the bathroom doors. To further enhance the realism of the environment, additional non-player avatars were placed throughout the environment at key locations (i.e., welcome desk, arcade machines, dining area), and directional background noise was integrated within the environment (e.g., arcade game music, air hockey puck, ambient bar clatter). Virtual mirrors lined the full length of the right-hand wall and the bathroom sink, allowing participants to view their avatars throughout the RPG. Additional scenes illustrating the virtual environment are displayed in Supplementary Material D. Notably, all non-player avatars were drawn from the broader pool of models developed by the research team for use in human–computer interaction research and were designed to represent cisgender individuals. Transgender characters were not included among the non-player avatars as the simulation was intended to reflect a predominantly cisgender environment, thereby positioning the transgender participant and colleague avatars as socially distinct within the interaction context.

To allow participants to move through the environment, blue teleportation rings were placed at key locations. The colleague avatar was programmed to walk to the next location before text-based instructions appeared to guide participants to each location in sequence (e.g., welcome desk, gaming tables, bar and dining area, bathrooms, lobby). Participants then navigated to the specified location by pointing their controller at a blue ring and selecting it. Using teleportation as the locomotion mechanic allowed participants to be instantly moved to the selected point in virtual space, without physically walking in the laboratory while wearing the VR headset.

#### Dialogue engine and scripting

In an RPG, a dialogue engine is a computational framework that facilitates interactive communication, narrative progression, and player agency by using *dialogue trees* and *scripting logic*. Dialogue trees refer to the hierarchical structure of predefined player dialogue options and resulting interaction partner responses, such that the player is permitted to choose their conversational path from a predetermined set of options, while the interaction partner’s response may differ depending on the option selected (i.e., branching narratives). Scripting logic then refers to the rules and conditions that dynamically adjust conversations based on the player’s choices. In the current study, participants were enabled to choose how they would like to respond to their colleague from the options presented within VR during conversational turn-taking. To enhance realism of the interaction, participants were instructed to speak their chosen dialogue aloud before selecting their response with their controller, which then cued the next scripted action (e.g., colleague dialogue, environment navigation, gameplay).

The content of the dialogue was designed to progressively increase in intimacy, such that participant-colleague interactions began with discussions surrounding the workplace and information technology skills, followed by discussions focused on likes and dislikes (e.g., food, pets), and then workplace equity, diversity, and inclusion. During the confrontation, participants were enabled to choose from a selection of responses to the aggressor that were modelled as passive or assertive. In addition to allowing the colleague to respond specifically to participant dialogue selections, our scripting logic adjusted the dialogue depending on the participant’s avatar gender (i.e., transgender woman/man) as well as the colleague’s gender (transgender/cisgender, woman/man). In particular, the colleague’s dialogue during the debrief was adjusted to represent lived experience for the transgender colleague versus allyship for the cisgender colleague. The full script and branching logic can be viewed in Supplementary Material E.

### Data processing

Postural sway timeseries were recorded across the six phases of the RPG by tracking the position of the head using the Vive headset, which captures linear motion trajectories along the X-, Y-, and Z-axes at 90 Hz (i.e., 90 captures per second). As postural sway was not recorded during gameplay, timeseries captured postural activity across the following periods of participant-colleague interaction: (1) from the start of the experiment to the start of the first game, (2) from the end of the first game to the start of the second game, (3) from the end of the second game to the start of the third game, (4) from the end of the third game to bathroom choice, (5) during the confrontation, (6) when debriefing with the colleague.

To prepare timeseries for DFA, we calculated a two-dimensional vector using data from the X- and Z-axes of linear motion. At baseline, the X-axis captures medial–lateral (M-L) sway, while the Z-axis captures anterior–posterior (A-P) sway. However, M-L and A-P sway were not independently constrained to these axes, as participants were permitted to rotate on the spot within the experimental space. That is, each phase of the RPG occurred at a different location within the virtual environment via teleportation, with the participant’s orientation rotated accordingly. To ensure that teleportation behaviours were excluded from postural activity data, each timeseries was trimmed by 1024 frames (i.e., 11.11 s) from the initial and final timestamps. Data processing was completed using Python (version 3.9.12), while DFA was completed using MATLAB (version R2024b). Example code can be viewed at: https://github.com/xkiwilabs/MATLAB-Toolboxes/tree/master/Fractal_Analysis_Toolbox.

### Detrended fluctuation analysis (DFA)

DFA is a non-linear timeseries method that determines the degree to which a behaviour or signal changes over time and across different timescales. DFA was conducted on each of the six trimmed timeseries, across all participants, by dividing each timeseries into segments of equal length varying by window size (i.e., length of non-overlapping segments comprised of 8, 16, 32, 64, 128, 256 data points). Within each segment, the local trend is identified and extracted from the data (i.e., detrended), isolating the residuals (i.e., differences between the data points and the local trend). The residual variance is then calculated for each segment, quantifying the degree to which the data fluctuates around the trend within that segment. Root mean square is provided as an estimate of residual variances averaged across segments for each window size. These residual variance estimates, representing the average magnitude of fluctuations in postural activity, are plotted as a function of window size in log–log form, with the slope of the regression line fitting this plot denoted as *α* (alpha). The scaling exponent *α* characterises the complexity of the fractal, or self-similar, structure within each timeseries^33,34^. This factor was extracted for each of the six postural sway timeseries and evaluated against standardised values representing white noise (*α* = 0.5; random, highly constrained variation), pink noise (*α* = 1.0; moderately persistent, highly flexible), and brown noise (*α* = 1.5; highly persistent, overly controlled).

### Measures

#### Embodiment

Perceived sense of virtual avatar embodiment was measured using the Embodiment Questionnaire^60^. Participants rated their experience of virtual avatar embodiment during the RPG on perceived body ownership, agency, self-location, and external appearance across 15 items from 1 (*strongly disagree*) to 7 (*strongly agree*). The Embodiment Questionnaire has previously demonstrated convergent, discriminant, and construct validity, with good internal consistency at α = 0.69-0.75^61^. In the current study, the embodiment questionnaire achieved an α of 0.65.

#### Gender essentialism

Endorsement of gender essentialism was measured using the Essential Sex and Gender subscale of the Heteronormative Attitudes and Beliefs Scale (HABS)^36^. Participants indicated the degree to which they endorsed eight statements concerning binary conceptualisations of gender from 1 (*strongly disagree*) to 7 (*strongly agree*). The HABS has previously demonstrated convergent and concurrent validity, with excellent internal consistency for this subscale at α = 0.85-0.92^36^. In the current study, the HABS achieved an α of 0.90.

#### Social desirability

The degree to which participants tended to respond to self-report measures in a favourable light was measured using the Marlowe-Crowne Social Desirability Scale (MCSDS)^62^. Participants responded to 33 true/false items concerning common, undesirable and unlikely, desirable behaviours. The MCSDS has previously demonstrated convergent and discriminant validity and good internal consistency at α = 0.70-0.80^63^. In the current study, the MCSDS achieved an α of 0.68.

#### Trait empathy

Trait-level empathetic tendencies were measured using the Questionnaire of Cognitive and Affective Empathy (QCAE)^64^. Participants responded to 19 items for cognitive empathy and eight items for affective empathy, excluding the Peripheral Responsivity subscale^65^ from 1 (*strongly disagree*) to 4 (*strongly agree*). The QCAE has previously demonstrated external, differential, and convergent validity, with good to excellent internal consistency (Cognitive: α = 0.86, Affective: α = 0.73)^65^. In the current study, we observed α = 0.82 for the Cognitive subscale and α = 0.68 for the Affective subscale.

#### State empathy

Context-specific empathy was measured at four timepoints during the RPG, using three items from Batson and colleagues^66^. Within VR, participants rated their feelings of compassion, warmth, and sympathy toward their colleague from 0 (*not at all*) to 10 (*very much*) following each game and after the debrief. This three-item measure has previously demonstrated convergent, discriminant, and predictive validity and excellent internal consistency at α = 0.85-0.88^67^. In the current study, we observed α = 0.81 at time 1, α = 0.85 at time 2, α = 0.86 at time 3, and α = 0.87 at time 4.

#### Interpersonal affiliation

Perceived likeability of and similarity with the colleague was measured pre- and post-RPG, using single item statements adapted from the Reysen Likeability Scale^38,68^. Participants were shown an image of their randomly assigned colleague’s avatar and provided ratings from 1 (*not at all*) to 9 (*very much*) for likeability (*How much do you like your colleague?*) and similarity (*How similar to you is your colleague?*).

## Supplementary Information

Below is the link to the electronic supplementary material.

Supplementary Material 1

## Data Availability

The de-identified dataset generated and analysed during this study is available at: https://doi.org/10.6084/m9.figshare.29288978.
