# Supplementary material for: Using a virtual reality role-playing game to enhance interaction dynamics and improve social attitudes toward transgender people
Source: Sci Rep. 2026 Apr 10;16:16943. doi: 10.1038/s41598-026-46402-3 (PMC13230618; doi:10.1038/s41598-026-46402-3)
Supplement: Supplementary file 1 — Supplementary Material 1 [file 41598_2026_46402_MOESM1_ESM.pdf]

Supplementary Material A

Colleague Avatar Vignettes

Transgender Man

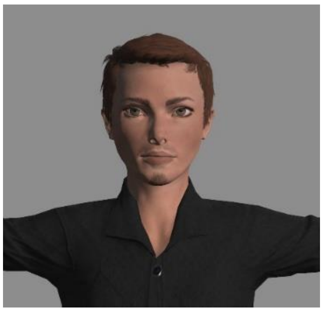

You will be meeting your colleague Alex today at the gaming centre GameZoid for a team-building event. Alex graduated from university and then worked in the information technology field as a programmer for five years before seeking new employment at your current company. He identifies as a *transgender man* and has a *masculine* appearance. Alex's pronouns are *he/him*.

Transgender Woman

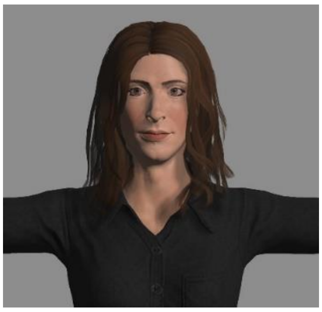

You will be meeting your colleague Alex today at the gaming centre GameZoid for a team-building event. Alex graduated from university and then worked in the information technology field as a programmer for five years before seeking new employment at your current company. She identifies as a *transgender woman* and has a *feminine* appearance. Alex's pronouns are *she/her*.

Cisgender Woman

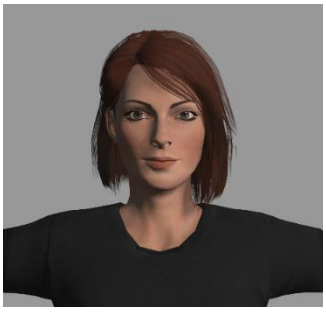

You will be meeting your colleague Alex today at the gaming centre GameZoid for a team-building event. Alex graduated from university and then worked in the information technology field as a programmer for five years before seeking new employment at your current company. She identifies as a *cisgender woman* and has a *feminine* appearance. Alex's pronouns are *she/her*.

Cisgender Man

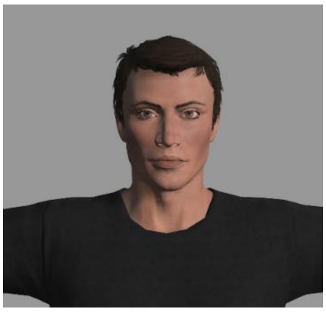

You will be meeting your colleague Alex today at the gaming centre GameZoid for a team-building event. Alex graduated from university and then worked in the information technology field as a programmer for five years before seeking new employment at your current company. He identifies as a *cisgender man* and has a *masculine* appearance. Alex's pronouns are *he/him*.

Note. Avatar profile descriptions were adapted from Dray et al. (2020).

Dray, K. K., Smith, V. R. E., Kostecki, T. P., Sabat, I. E., & Thomson, C. R. (2020). Moving beyond the gender binary: Examining workplace perceptions of nonbinary and transgender employees. *Gender, Work & Organization*, 1-11. https://doi.org/10.1111/gwao.12455

Supplementary Material B

Pre-RPG Experimenter Instructions and Movement Integration

Avatar Identity Reinforcement

In a few moments, you will be in an area that looks like the lobby of the gaming centre, and you should be able to see yourself in the mirror in front of you.

You should be able to see your avatar in the mirror now. Please stand with your arms resting at your sides and looking straight ahead.

Could you confirm whether you are the first or the second avatar you created?

You've been randomly assigned to be the transgender avatar that you created. I'm now going to walk you through a brief movement exercise to get better acquainted with the virtual experience.

Movement Integration Exercise

1) Go ahead and take a few moments to move around with your head and arms freely.
2) Now, take a few moments to view yourself in the mirror in front of you.
3) When you're ready, turn your head and look to the left.
4) Look back to the centre.
5) Then turn your head and look to the right.
6) Look back to the centre.
7) Look up.
8) And then look down.
9) Now look straight ahead.
10) While holding the controller, move your right arm and hold it out to the side.
11) Move your extended arm all the way up.
12) And then all the way down to your side.
13) We'll now do the same with the left arm. Please hold it out to the side.
14) Move your extended arm all the way up.
15) And then all the way down to your side.
16) To finish we'll do both arms at the same time.
17) Hold both arms out to the side.
18) Move both arms all the way up.
19) And then all the way down to rest.

Supplementary Material C

Additional Virtual Avatars

Selection of Customised Participant Avatars

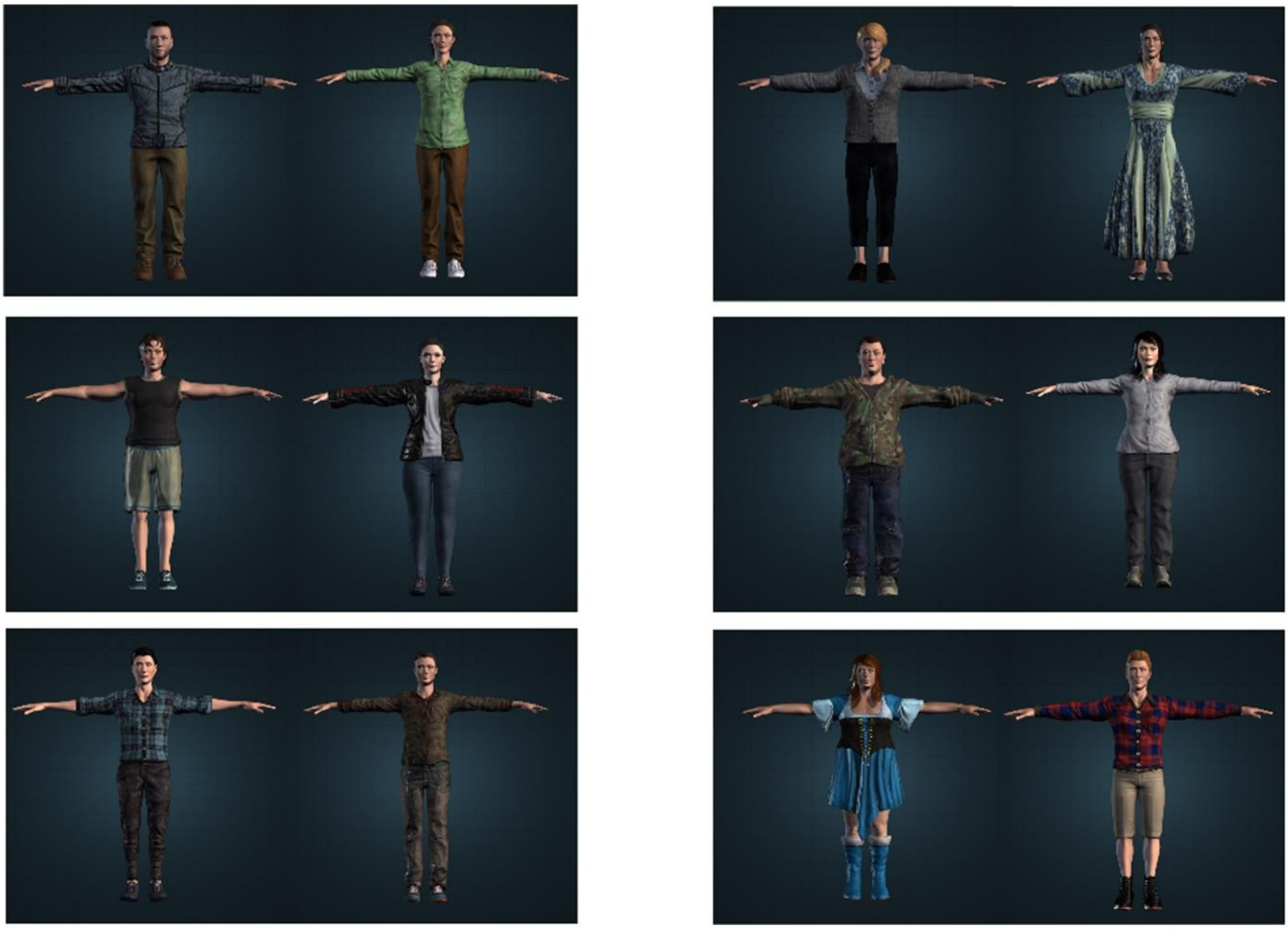

Aggressor Avatars

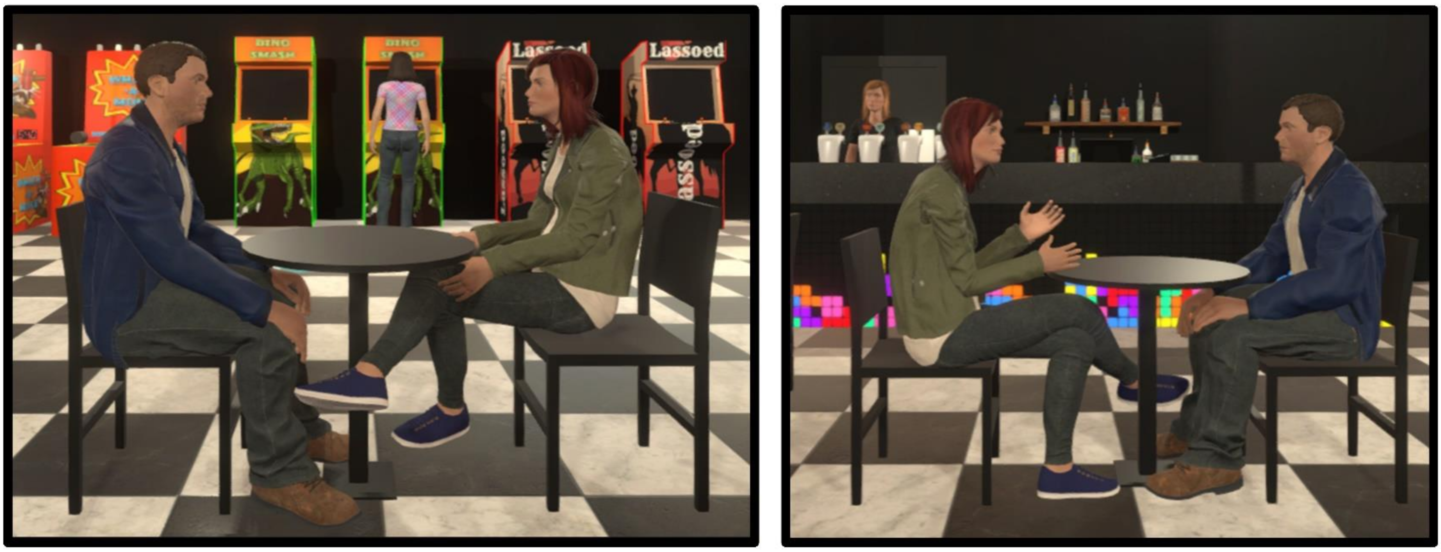

Supplementary Material D

Additional Virtual Scenes

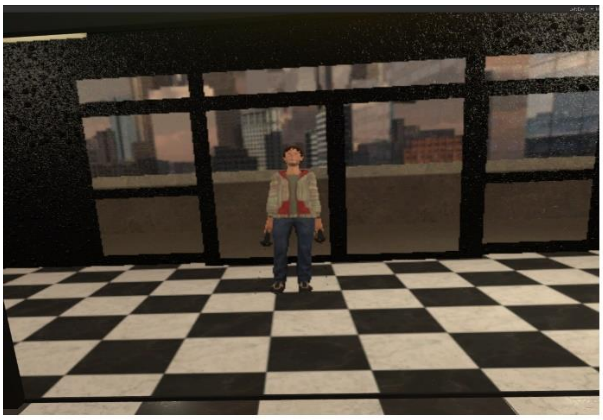

Lobby and Virtual Mirror

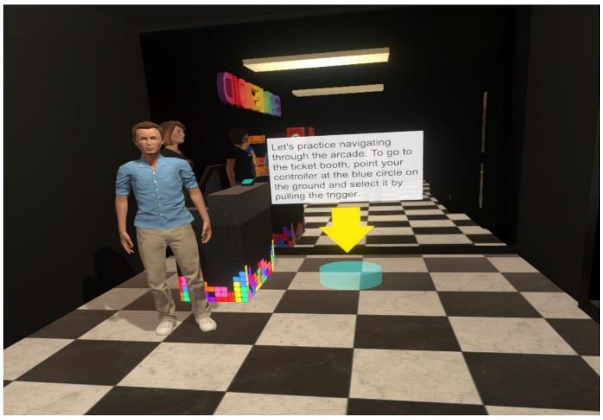

Welcome Desk

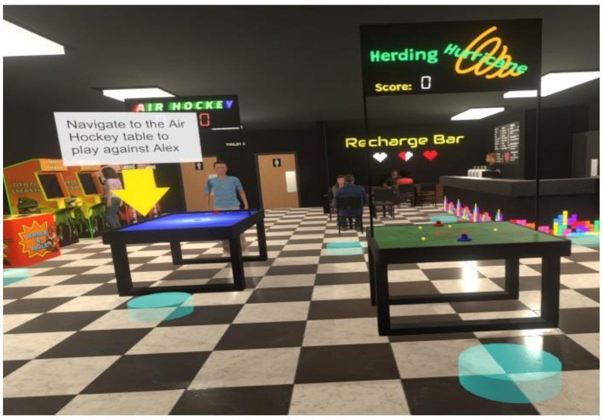

Central Gaming Area

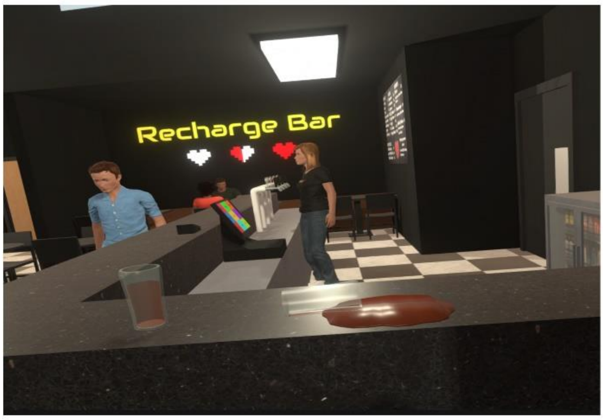

Bar and Dining Area

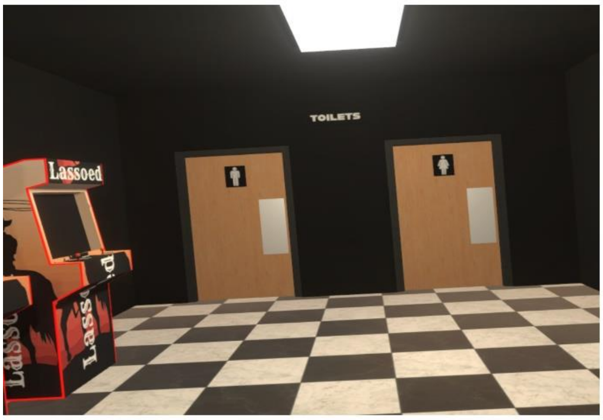

Bathrooms (External)

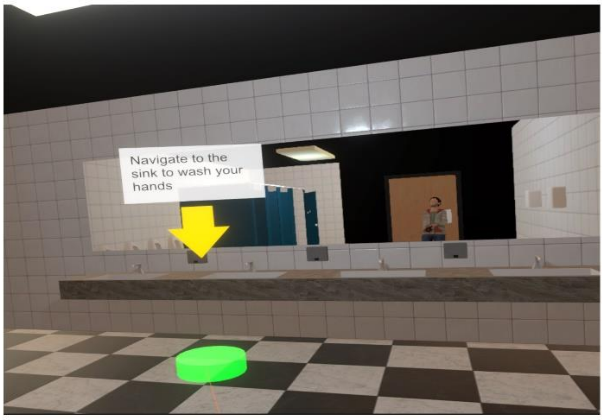

Bathrooms (Internal)

Supplementary Material E

Dialogue Engine Script and Branching

Note. Upright text is used to indicate the colleague's dialogue, while italicised text denotes participant response options.

Phase 1

Hi, I'm Alex. My pronouns are [she/her, he/him]. Thanks so much for joining me today for this month's social event!

> *Hi Alex, it's nice to meet you. I'm Sam and my pronouns are [she/her, he/him].*

Very nice to meet you Sam. So, I'm in a project manager in the IT department and the boss tells me you're in IT as well. What do you do?

> *I'm a programmer.*
>
> *I'm a software developer.*
>
> *I'm a data analyst.*

Oh awesome! Well, welcome to the company. It's great to have some new talent on board, and I'm sure we'll be working on a project together soon. How are you feeling about that?

> *Actually, I'm a little bit nervous being the new [girl/guy].*
>
>> I understand. I was nervous when I was the new [girl/guy] too.
>
> *Hmm, I'm not sure exactly what to expect yet. But that's how it is when you're the new [girl/guy].*
>
>> I remember feeling that way when I was the new [girl/guy] too.
>
> *Yeah, I'm feeling really good about working together. Thanks for welcoming me as the new [girl/guy].*
>
>> That's great to hear! I know what it's like to be the new [girl/guy] too.

And that's exactly why we do these events! What today is all about is getting you a little bit more comfortable with your new team outside of the office. So, shall we get started with the activities?

> *Yes, let's do it!*
>
> *Hmm, maybe in a minute. I'd like to chat a bit more first.*
>
>> Ok, no worries. What's on your mind?
>>
>>> *How long have you been at the company?*
>>>
>>>> Oh, it's been about five years now.
>>>
>>> *How many people do you usually work with on a project?*
>>>
>>>> It varies, but usually around six to ten.

We'll definitely get to know some more about each other while we play some games today. The boss has given us some passes, so let's go get our tickets at the counter.

Okay, it looks like we've got enough to play three games and then grab some food and drink. Let's go see what games there are.

> *I'm not much of a gamer. Which game do you think we should try first?*

I’ve only played a few games before. Which game do you think we should try first?

I play a lot of games, but I’ve never been here before. Which game do you think we should try first?

Okay. I’ve been here before, so let’s start with this one.

Phase 2

Good work, Sam! How did you like the first game?

(IF HERDING)

I think it was great! It’s nice to play as a team.

Awesome! I really enjoyed playing with you as part of a team.

I think it was okay, but I found it a bit challenging.

Yeah, it is a bit of a challenge, isn’t it? But I really enjoyed playing with you as part of a team!

I didn’t like it very much. I prefer games where there’s a little competition.

Competitive games are great too, but I really enjoyed playing with you as part of a team.

(IF AIR HOCKEY = WIN)

I liked it a lot, and winning is definitely a bonus!

It was okay, but winning is definitely a bonus!

I didn’t like it very much, but winning is definitely a bonus!

Winning is certainly a bonus. Good job on your win, Sam. You were a tough competitor!

(IF AIR HOCKEY = LOSE)

I liked it a lot, even though I lost. But good job on your win!

It was okay, even though I lost. But good job on your win!

I didn’t like it very much, and I lost. But good job on your win!

Thanks, Sam. But you were a tough competitor too!

Before we play the next game, let’s get to know each other a little more. Let’s see. Do you have any pets at home?

Yes, I have a dog. How about you?

Yes, I have a cat. How about you?

Yes, I have a _____ (INSERT ANIMAL HERE). How about you?

No, I don’t have any pets, but I’d like to someday. How about you?

No, I don’t have any pets, I prefer plants. How about you?

I have a cat and a dog, and some plants too! It's nice to come home from work to see them every day. Now your turn. What would you like to know about me?

What’s your favourite food?

Well, it’s hard to pick a favourite, but a go to is always pizza! Maybe we can get some later at the bar.

Yes, that sounds good.

Okay, but I might have something else instead.

Do you have any special talents?

Well, I do like to sing. Karaoke is always fun. Maybe we can do that at the next team-building event!

Yes, that sounds like a great idea.

Okay, but I'm not very good at karaoke!

Sounds like a plan! Thanks for sharing, Sam. Now, let's go play the next game.

Phase 3

That was fun! Have you played this game before?

No, I haven't.

Yes, I have.

Well, you did a good job. It's definitely a challenge to get all the moles! Let's take a little break before the third game. Tell me a bit more about yourself. What skill do you think everyone should learn?

Well, Alex, in our field, I think everyone should learn about customer service.

Well, Alex, in our field, I think everyone should learn about digital marketing.

Well, Alex, in our field, I think everyone should learn about problem solving.

Yes, I totally agree. That's very important in IT, and definitely something we will work on together in the office! Would you like to know anything else about our team?

Yes, I'd like to know about diversity and inclusion.

Of course! Diversity and inclusion are very important on our team. Everyone makes a unique contribution, and we respect differences between people. Integrating diverse perspectives is just one of the things that makes our team work so well together.

Yes, I'd like to know about allyship.

Of course! Allyship is very important on our team. It means that we can recognise social privileges alongside others' experiences. We can then support our fellow colleagues through advocacy and self-reflection.

Yes, I'd like to know about gender visibility.

Of course! Gender visibility is very important on our team. Everyone should feel comfortable, valued, and respected. One of the ways we do this is by actively sharing pronouns and correcting any assumptions.

That was a great question. As a project manager, my door is always open for these conversations, and you can come talk to me about it any time. This kind of company culture is why I like working here, and I hope you will too. Okay Sam, I think it's time for the last game, and then we'll go grab some food!

Phase 4

Well, I think that's the last game. How was it for you?

[DIALOG AS ABOVE – IF HERDING/IF AIR HOCKEY = WIN/IF AIR HOCKEY = LOSE]

That was a lot of fun, great game! I've worked up an appetite now. Should we head over to the bar?

Yes, let's go.

Let's see, I'll have a drink and some pizza. What would you like, Sam?

I'll have pizza too, and a soda, please.

I'll just have a soda today. I'm not very hungry.

I'll have the nachos and a soda, please.

Great, let's grab our drinks and have a seat while we wait for the food.

[DRINK SPILLS]

Oh no! That's bad luck. Why don't I grab you a refill and you can head over to the bathrooms to wash off your hands.

Phase 5 *(Note.* Upright text indicates the aggressor avatar's dialogue during this phase.)

Hey! I saw that you were just in the [women's/men's] bathroom. Why were you in there?

I spilled my drink, so I went to wash my hands.

Yea ok, but you were obviously in the wrong bathroom!

No, I don't think I was.

Yes, you were. You belong in the [men's/women's] room!

Okay, well, I'd really like to avoid a conflict here. So, I apologise, but that is where I am most comfortable as a transgender [woman/man].

Actually, I'm a transgender [woman/man], and I didn't do anything wrong by being in that bathroom. That's where I'm most comfortable.

Oh, okay. Uh sorry about that.

Yea, you should be sorry. You belong in the [men's/women's] room!

Okay, well, I'd really like to avoid a conflict here. So, I apologise, but that is where I am most comfortable as a transgender [woman/man].

Actually, I'm not sorry. I'm a transgender [woman/man], and I didn't do anything wrong by being in that bathroom. That's where I'm most comfortable.

Well, I don't think that's okay. It's completely inappropriate and makes everyone feel unsafe.

Okay, I guess I can see where you're coming from, although your opinion is very conservative. I'm going to go now. My colleague is over there.

Actually, I think you are making people feel unsafe. Your opinion is very ignorant. I'm going to go now. My colleague is over there.

Phase 6

Hey, Sam. I saw that person seemed to be arguing with you what happened?

They told me I was using the wrong bathroom. I didn't want to argue though. That would be too hard, so I left.

They told me I was using the wrong bathroom. They weren't listening to my arguments. I felt very unsafe, so I left.

(IF TRANSGENDER COLLEAGUE)

That's terrible! That's happened to me before too. How are you feeling?

I'm feeling very sad.

I definitely remember that feeling. I'm sorry that happened to you.

I'm feeling a bit angry.

I am angry too hearing about it. I'm sorry that happened to you.

I'm feeling okay now.

I'm glad you feel better after leaving. I'm sorry that happened to you.

It's good to talk to someone who understands! I really appreciate it.

Thanks for listening. I'm feeling a bit better now.

(IF CISGENDER COLLEAGUE)

That's terrible! I can't say I've had that experience, but how are you feeling?

I'm feeling very sad.

I'm feeling a bit angry.

I'm feeling okay now.

I can understand how you would feel that way. I'm sorry that happened to you.

Thanks for listening. I really appreciate it.

Thanks for trying to understand. I really appreciate it.

(END FOR BOTH COLLEAGUES)

No worries, Sam. If you'd like to talk more about this back in the office, my door is always open. Before we go, would you like me to file a report about the incident?

No, that's okay.

Okay, let me know if you change your mind.

Yes, I think that's a good idea.

Okay, I'll do that as soon as possible.

Well, Sam, I think that's the end of today's activities. Thank you so much for joining me today, and I hope you had a good time, despite the incident. It's been great getting to know you a bit, and I look forward to working together more!

Thanks, Alex. It's been great getting to know you too. I'll see you around the office!
